# LOX-1 mediates inflammatory activation of microglial cells through the p38-MAPK/NF-κB pathways under hypoxic-ischemic conditions

**DOI:** 10.1186/s12964-023-01048-w

**Published:** 2023-06-02

**Authors:** Yoshinori Aoki, Hongmei Dai, Fumika Furuta, Tomohisa Akamatsu, Takuya Oshima, Naoto Takahashi, Yu-ichi Goto, Akira Oka, Masayuki Itoh

**Affiliations:** 1 Department of Mental Retardation and Birth Defect Research, National Institute of Neurology, National Center of Neurology and Psychiatry, Kodaira, Tokyo, Japan; 2 Department of Pediatrics, Graduate School of Medicine, The University of Tokyo, Tokyo, Japan; 3 Present Address: Department of Pediatrics, Faculty of Medicine, University of Miyazaki, Kiyotake-cho Kihara 5200, Miyazaki, 889-1692 Japan; 4 Present Address: Department of Pediatrics, Center Hospital of the National Center for Global Health and Medicine, 1-21-1 Toyama, Shinjuku-ku, Tokyo, Japan; 5 Present Address: Saitama Children’s Medical Center, 1-2 Shintoshin, Chuo-ku, Saitama, Japan

**Keywords:** Hypoxia, Ischemia, Microglia, p38-MAPK, NF‐kappa B (NF‐κB), *OLR-1*, LOX-1

## Abstract

**Background:**

Microglial cells play an important role in the immune system in the brain. Activated microglial cells are not only injurious but also neuroprotective. We confirmed marked lectin-like oxidized low-density lipoprotein receptor-1 (LOX-1) expression in microglial cells in pathological lesions in the neonatal hypoxic-ischemic encephalopathy (nHIE) model brain. LOX-1 is known to be an activator of cytokines and chemokines through intracellular pathways. Here, we investigated a novel role of LOX-1 and the molecular mechanism of LOX-1 gene transcription microglial cells under hypoxic and ischemic conditions.

**Methods:**

We isolated primary rat microglial cells from 3-day-old rat brains and confirmed that the isolated cells showed more than 98% Iba-1 positivity with immunocytochemistry. We treated primary rat microglial cells with oxygen glucose deprivation (OGD) as an in vitro model of nHIE. Then, we evaluated the expression levels of LOX-1, cytokines and chemokines in cells treated with or without siRNA and inhibitors compared with those of cells that did not receive OGD-treatment. To confirm transcription factor binding to the *OLR-1* gene promoter under the OGD conditions, we performed a luciferase reporter assay and chromatin immunoprecipitation assay. In addition, we analyzed reactive oxygen species and cell viability.

**Results:**

We found that defects in oxygen and nutrition induced LOX-1 expression and led to the production of inflammatory mediators, such as the cytokines IL-1β, IL-6 and TNF-α; the chemokines CCL2, CCL5 and CCL3; and reactive oxygen/nitrogen species. Then, the LOX-1 signal transduction pathway was blocked by inhibitors, LOX-1 siRNA, the p38-MAPK inhibitor SB203580 and the NF-κB inhibitor BAY11-7082 suppressed the production of inflammatory mediators. We found that NF-κB and HIF-1α bind to the promoter region of the *OLR-1* gene. Based on the results of the luciferase reporter assay, NF-κB has strong transcriptional activity. Moreover, we demonstrated that LOX-1 in microglial cells was autonomously overexpressed by positive feedback of the intracellular LOX-1 pathway.

**Conclusion:**

The hypoxic/ischemic conditions of microglial cells induced LOX-1 expression and activated the immune system. LOX-1 and its related molecules or chemicals may be major therapeutic candidates.

**Video abstract**

**Supplementary Information:**

The online version contains supplementary material available at 10.1186/s12964-023-01048-w.

## Background

Microglial cells are the resident immune cells of the central nervous system (CNS) and have diverse functions that are both beneficial and harmful [1–3]. It has been reported that pathological conditions in CNS disease model animals deteriorate when microglial cells are depleted or do not function properly, as well as that microglial cells help model animals recover from illness [4, 5]. However, the other studies have reported that blocking microglial activities improves that disease phenotype [6, 7]. The function of activated microglial cells is not only injurious but also neuroprotective because activated microglial cells can release anti-inflammatory cytokines and trophic factors [1–3]. Activated microglial cells are classified into two major phenotypes, the M1 phenotype (inflammatory phenotype) and the M2 phenotype (anti-inflammatory phenotype) [8–10]. Several studies have focused on these microglial phenotypes in a variety of CNS diseases [11–14]. Microglial cells may contribute to the pathology of the premature brain. In the premature brain, perinatal inflammation mediated by microglia contributes significantly to neurodevelopmental injuries including white matter injury [15–19]. Periventricular leukomalacia, which is the most common cause of cerebral palsy, is caused by hypoxia ischemia affecting premature brain and characterized by white matter necrotic lesions, microglial activation, NF-κB induction and neuronal death [20].

Neonatal hypoxic-ischemic encephalopathy (nHIE) is a major cause of perinatal brain damage that is thought to occur via multiple events such as placental abruption, umbilical cord prolapses, or maternal/fetal infection, ultimately inducing an inadequate supply of oxygen and blood to the brain [21, 22]. The incidence of nHIE is 1–8 per 1000 live births in developed countries and is much higher in undeveloped countries [23]. Despite many animal experiments and clinical trials, the pathophysiology is still not clear and an effective therapy has yet to be established [24, 25]. We have reported that lectin-like oxidized low-density lipoprotein receptor-1 (LOX-1), the gene named *oxidized low-density lipoprotein receptor 1* (*OLR1*), was upregulated in the nHIE rat brain and administration of an anti-LOX-1 neutralizing antibody improved brain pathology [26]. LOX-1 is a scavenger receptor for modified proteins, especially oxidized low-density lipoprotein (oxLDL) [27]. LOX-1 is expressed on endothelial cells, smooth muscle cells and macrophages and is related to the development of atherosclerosis and other cardiovascular diseases. In addition, LOX-1 acts physiologically as an inducer of apoptosis, reactive oxygen species and proinflammatory cytokines [28–30]. We also found that LOX-1 was expressed in microglial cells, the resident brain macrophages, in the nHIE rat model [31]. In addition to other CNS diseases, it is generally recognized that inflammation induced by the peripheral and central immune systems is one of the major pathological factors in nHIE [32–34]. Although microglial cells are thought to be related to the pathology of nHIE, much remains unknown regarding the role of microglial cells in nHIE and the therapeutic strategy targeting microglial cells. In addition, *Olr1* transcription is regulated by NF-κB, HIF-1α and OCT-1 [35–37]. It is well known that hypoxic conditions accelerate HIF-1-dependent transcription. Moreover, it is reported that inflammatory reactions directly affect redox-sensitive transcription factors of NF-κB or Oct-1 and indirectly modulate activity or stability of HIF-1 or the inhibitors of NF-κB [38]. However, the relationship of those transcription factors and LOX-1 has been never known.

We hypothesized that LOX-1 mediates the inflammatory activation of microglial cells under hypoxic/ischemic conditions, which induces neuronal death. In the present study, we investigated the alteration in LOX-1 expression in microglia, which is controlled by specific transcription factors and the influence of LOX-1 on detrimental processes under hypoxic/ischemic conditions using primary rat microglial cell cultures. Furthermore, we explored the signaling pathways related to LOX-1 in microglia to establish a new treatment strategy for nHIE.

## Methods

### Primary microglial cell isolation from newborn rat brains

We obtained primary microglial cells according to a previously described technique [39, 40]. Briefly, mixed glial cultures were prepared from newborn to 3-day-old Sprague–Dawley rat brains containing the cortex, hippocampus and striatum (CLEA Japan Inc., Tokyo, Japan) and cultured until confluent. The cells were cultured in high-glucose Dulbecco’s modified Eagle medium (DMEM high glucose, WAKO, Osaka, Japan) containing 10% fetal bovine serum (FBS) and penicillin/streptomycin at 37 °C in a 10% CO_2_ incubator. After approximately 2 weeks, microglial cells were harvested by the shaking method and replated on 6-well plates at 2 × 10^6^ cells/well, 96-well plates at 1 × 10^5^ cells/well or 8-well chamber slides at 2 × 10^5^ cells/well. The microglial cell cultures consisted of > 98% microglial cells (stained with anti-Iba1 antibody (WAKO)) (Additional file 4: Fig. S1). All animal experiments were performed with the permission of the Animal Experiment Ethics Committee of the National Center of Neurology and Psychiatry.

### Oxygen glucose deprivation and LOX-1 knockdown treatments

We performed oxygen glucose deprivation (OGD) treatment as an in vitro model of hypoxia/ischemia [41, 42]. The harvested microglial cells were cultured in high-glucose DMEM containing 10% FBS for 24 h. Then, the culture medium was replaced with glucose-free DMEM without FBS. Primary microglial cells were treated with OGD and LOX-1 knockdown.

Regarding OGD treatment, cultured microglial cells were treated with a BIONIX-2 hypoxic cell culture kit (Sugiyamagen, Tokyo, Japan). The concentration of oxygen was maintained at almost 0%. For the controls, the culture medium was replaced with high-glucose DMEM without FBS and microglial cells were cultured under normal oxygen concentrations. After OGD treatment for 6 h, the microglial cells were washed with PBS. Then, total RNA or proteins were extracted for analysis and the culture supernatant was collected for cytokine measurement. For immunocytochemistry, the cells in chamber slides were washed with PBS and then fixed with 4% paraformaldehyde (PFA).

For LOX-1 knockdown, the expression of LOX-1 in primary microglial cells was silenced using the small interfering RNA (siRNA) Silencer Select rat *Olr1* (gene name of LOX-1) (Thermo Fisher Scientific, Waltham, MA) with Lipofectamine RNAiMAX transfection reagent (Thermo Fisher Scientific) according to the manufacturer’s instructions. We used Silencer Select negative control No.1 siRNA that did not target any rat genes as a negative control. The diluted siRNA (12.5 pmol/ml) and transfection reagent (3.75 μl/ml) added to the microglial cell culture. The cells were incubated with the siRNA for 24 h and then were subjected to the OGD experiments.

### Inhibitors of p38-MAPK and NF-κB

To investigate whether OGD affected LOX-1 pathway, we used two inhibitors of p38-MAPK and NF-κB. SB203580 and BAY11-7082 are frequently used in in vitro study. SB203580 (199-16551; Wako Pure Chemical Industries, Osaka, Japan), a p38-MAPK inhibitor, was added to cultured microglial cells at a concentration of 20 μmol/L for 60 min before OGD treatment [43]. BAY11-7082 (19542-67-7; Wako Pure Chemical Industries), an NF-κB inhibitor, was added to the isolated microglial cells at a concentration of 10 μmol/L for 30 min before OGD treatment [44].

### RNA extraction and real-time quantitative PCR

Total RNA was extracted from rat brains and cultured microglial cells using the RNeasy Plus Mini kit (Qiagen, Venlo, Netherlands) according to the manufacturer’s protocols. Then, the total RNA concentration was measured using a Nanodrop (Thermo Fisher Scientific). For RT-PCR, cDNA was prepared from total RNA using a high-capacity cDNA Reverse Transcription kit (Thermo Fisher Scientific). RT-PCR was performed using LightCycler 480 SYBR Green I master mix (Roche, Basel, Switzerland) on a LightCycler 480 System II (Roche). The experiments were performed in triplicate. The mRNA levels were normalized relative to the endogenous reference gene *act-b* (β-actin). The results are described as the fold change of the Ct value relative to the control groups. We used the primer sequences of *Olr1*: forward 5′-TGACCCTGCCATGCCATGCT-3′ and reverse 5′-TGGGGATGGTGGAGGCCCTG-3′, and *β-actin*: forward 5′-TAAGGCCAACCGTGAAAAGA-3′ and reverse 5′-GAGGCATACAGGGACAACACA-3′.

### Transcriptome analysis

Whole transcriptome microarray analysis was performed using a Rat Clariom S assay (Thermo Fisher Scientific) according to the manufacturer’s instructions. Analysis and normalization of the raw data were conducted using Transcriptome Analysis Console (TAC) 4.0 (Thermo Fisher Scientific). Differentially expressed genes (DEGs) were defined as genes with an FDR < 0.01 and a more than twofold change. Gene ontology (GO) and KEGG pathway analyses were conducted using GSEA software (http://software.broadinstitute.org/gsea/index.jsp).

### Protein extraction and immunoblotting

Total proteins were extracted using RIPA buffer (Nacalai Tesque, Kyoto, Japan) according to the manufacturer’s instructions. Separate cytoplasmic and nuclear protein fractions were extracted using NE-PE nuclear and cytoplasmic extraction reagents (Thermo Fisher Scientific). Then, the protein concentrations were measured using a Pierce BCA protein assay kit (Thermo Fisher Scientific).

Twenty micrograms of the extracted proteins were loaded in each lane of TruPAG Precast Gels 4–12% (Sigma-Aldrich Corporate, St. Louis, MO) for electrophoresis. After transfer to a polyvinylidine difluoride membrane, each immunoreacted band was detected using Amersham ECL prime Western blotting detection reagent (GE Healthcare, Boston, MA) according to the manufacturer’s instructions. We used primary antibodies against LOX-1 (AF1798; R&D Systems, Minneapolis, MN), NF-κB p65 (PA5-16545; Thermo Fisher Scientific), phospho-p38 MAPK (4511; Cell Signaling Technology Inc., Danvers, MA), p-38 MAPK (8690; Cell Signaling Technology Inc.), phosphor-ERK 1/2 (4370; Cell Signaling Technology Inc.) and ERK 1/2 (4695; Cell Signaling Technology Inc.), as previously described [26].

As a reference, β-actin (A5316; Sigma-Aldrich Corporate) and PCNA (2586; Cell Signaling Technology Inc.) were detected using a specific antibody. The expression levels of the detected bands were measured and calculated by ImageQuant TL (GE Healthcare).

### Immunocytochemistry

The microglial cells on chamber slides or in 96-well plates were washed with PBS and fixed with 4% PFA for 30 min. Then, the cells were incubated with the same primary antibodies used in immunoblotting analyses. Then, the cells were incubated with Alexa Flour-conjugated secondary antibodies (Thermo Fisher Scientific) for 1 h and mounted with Hoechst 33342 (Thermo Fisher Scientific). The fluorescent samples were observed with confocal laser scanning microscopy (LSM780; Zeiss, Oberkochen, Germany) or fluorescence microscopy (IX71; Olympus, Tokyo, Japan).

### Cytokine measurement

The microglial cell culture medium was collected and centrifuged for 20 min at 1000× *g*. Then, cytokine/chemokine concentrations in the supernatants were measured using a BioPlex ProTM Rat Cytokine 23-Plex Assay (Bio-Rad, Hercules, CA).

### Reactive oxygen species (ROS) detection

After the OGD experiments, microglial cells were subjected to OGD treatment and treated with 4.5% glucose containing original medium as controls. Then, cellular ROS levels were detected using CellRox Green Reagent (C10444: Thermo Fisher Scientific). Briefly, the microglial cells were stained with 5 μM CellRox Green Reagent by adding the probe to the complete media and incubating at 37 °C for 30 min. Fifty micromolar of *N*-acetyl cysteine (NAC), an antioxidant was added to some of the OGD-treated wells. Then, the cells were washed with PBS and fixed with 4% PFA. Finally, the microglial nuclei were stained with Hoechst 33342 (Roche). The cells were observed with a microscope (IX71: Olympus, Tokyo, Japan), and the mean fluorescent intensities per cell were analyzed using an In Cell Analyzer 2000 (GE Healthcare).

### Luciferase reporter assay

To construct the pGL3-WT luciferase reporter vector, the 2372-nt fragment (− 2336 to + 36) of human *OLR1* gene containing the binding sites for NF-κB, OCT-1 and HIF-1α was amplified by PCR using the following KpnI/MluI site-linked primers: forward 5′-ATCTGGGTACCTCAGTGTGATATCGTTTCAG-3′ and reverse 5′-TAGCTAGATCTTCTCTCCGAGAGGAGGGAGC-3′, and was cloned into the pGL3-Basic firefly luciferase reporter vector (Promega, Madison, WI). Site-directed mutagenesis of the putative NF-kB target site (GGGAGTCTC), OCT-1 target site (ACGCGT) and HIF-1α target site (A/GCGTC) with the pGL3- WT vector as the template was performed using the MutanBEST kit (D401; Takara) according to the manufacturer's instructions, and the result was called the PGL3-Mutant. The primer sets for the luciferase assay showed in Additional file 1: Table S1. All plasmid DNAs were extracted and purified with a Plasmid Midi Kit (Qiagen) and sequenced with the 3130 GeneAnalyser (Thermo Fisher Scientific).

The pGL3-WT and pGL3-Mutant luciferase reporter vectors were transfected into HEK293T cells using Lipofectamine 2000 Transfection Reagent (11668027; Thermo Fisher Scientific). Cells transfected with the pGL3-empty vector were used as controls. Cells were lysed 24 h posttransfection in passive lysis buffer (Promega). Firefly and Renilla luciferase signals were measured by the Dual-Luciferase Reporter Assay System (Promega) on a Centro Microplate Luminometer LB960 (Berthold Technologies, Baden Württemberg, Germany). Three biological replicates were performed for each sample in luciferase assays. Normalized reporter activity was expressed as the firefly luciferase value divided by the Renilla luciferase value (RLA), which was then normalized to the control vector activity.

### Chromatin immunoprecipitation assay

We identified the binding sites for NF-kB, Oct-1 and HIF-1α in the promoter region of the human *OLR1* gene from the UCSC Genome Browser (http://genome.ucsc.edu). To identify whether the transcription factors NF-κB and HIF-1α control the expression of LOX-1 in microglia, we performed a chromatin immunoprecipitation assay using the ChIP Kit-One Step (ab117138; Abcam, Cambridge, UK) with the HMC3 human microglial cell line (CRL-3304; American Type Culture Collection, Manassas, VA). According to the manufacturer’s instructions, we collected OGD-treated microglial cells, fixed them with 1% formaldehyde and fragmented them with sonication. We used antibodies against NF-κB (PA5-16545; Thermo Fisher Scientific) and HIF-1α (AF1935; R&D Systems) and primer sets for NF-κB and HIF-1α (Additional file 3: Table S2).

### Cell viability

After the OGD experiments, the microglial cells subjected to OGD treatment were treated with 4.5% glucose at the same glucose concentrations as the CTLs. Then, the cells were incubated for 2 h with CellTiter 96 AQueous One Solution Cell Proliferation Assay (Promega, Madison, WA) at 37 °C according to the manufacturer’s instructions. This reagent contains a novel tetrazolium compound [3-(4,5-dimethylthiazol-2-yl)-5-(3-carboxymethoxyphenyl)-2-(4-sulfophenyl)-2H-tetrazolium, inner salt; MTS]. The quantity of formazan product as measured by absorbance at 490 nm is directly proportional to the number of living cells in cultures. After 2 h of incubation, the absorbance at 490 nm was measured using a 96-well plate reader.

### Statistical analysis

All data are described as the means ± SEMs. Data were analyzed by one-way ANOVA followed by Tukey’s post hoc test or an unpaired *t*-test. Statistical analysis was performed using IBM SPSS version 22 (IBM, Armonk, NY). *P* < 0.05 was considered to be statistically significant.

## Results

### LOX-1, cytokine and chemokine expression and ROS induction in OGD-treated microglial cells

**Fig. 1:**
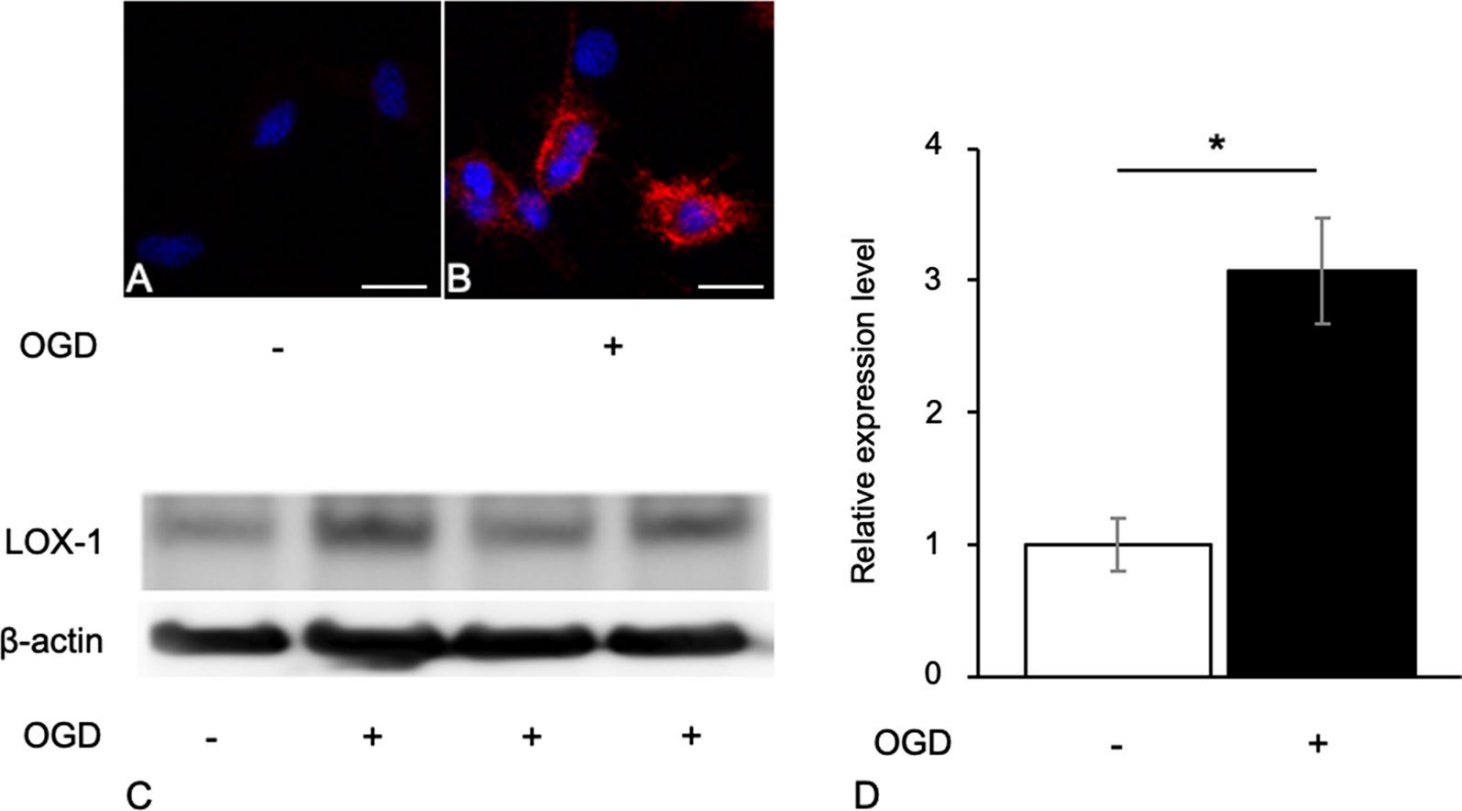
OGD-treated microglial cells express LOX-1. Untreated microglial cells (control; CTL) did not exhibit LOX-1 expression (**A**). OGD-treated microglial cells (OGD) exhibited LOX-1 expression in the cytoplasm (**B**). Immunoblot analysis shows LOX-1 overexpression in OGD-treated microglial cells (**C**, **D**). Immunocytochemistry, blue: Hoechst 33342 (**A**, **B**), red; LOX-1 (**B**). *P < 0.05. Scale bar = 10 µm

First, we confirmed that the cultured cells in all experiments were more than 98% Iba-1 positive (Additional file 4: Fig. S1). Many OGD-treated microglial cells had LOX-1 signals in the cytoplasm, although the untreated microglial cells had no LOX-1 signals (Fig. 1A, B).

**Fig. 2:**
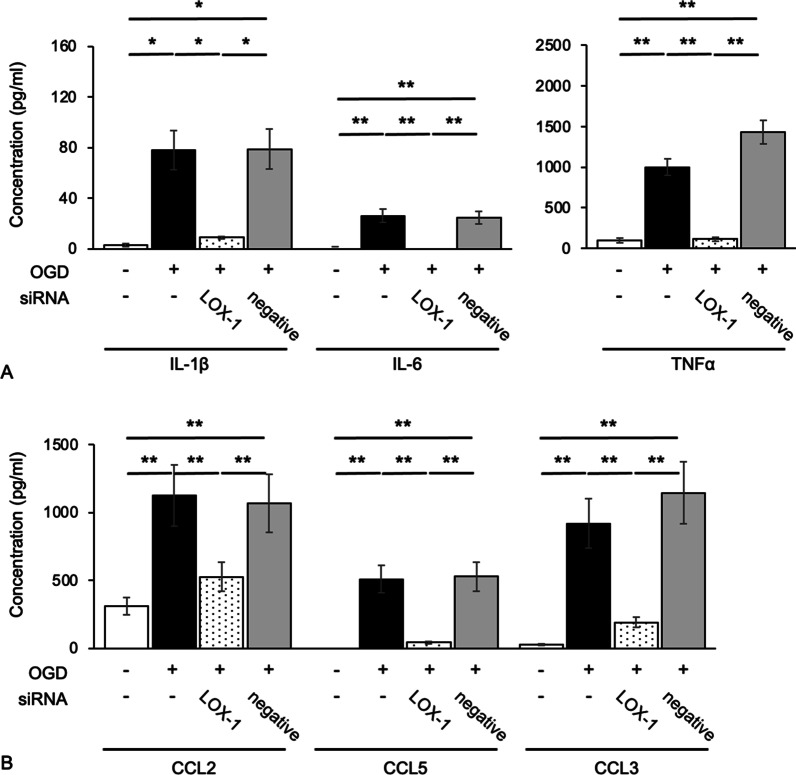
LOX-1 siRNA inhibits OGD-induced cytokine and chemokine production. The cytokines IL-1β, IL-6 and TNF-α (**A**) and the chemokines CCL2, CCL5 and CCL3 (**B**) were significantly increased. LOX-1 siRNA downregulates the expression of these molecules. **P < 0.01

Immunoblotting experiments showed that the expression of LOX-1 was increased in OGD-treated microglial cells compared with control cells (Fig. 1C, D). RT-PCR showed that the mRNA levels of LOX-1 were similar to those of the protein levels (Additional file 5: Fig. S2). Thus, the expression of LOX-1 in microglial cells was upregulated under hypoxic and ischemic conditions. OGD treatment also markedly induced the expression levels of the cytokines IL-1β, IL-6 and TNF-α and the chemokines CCL2, CCL5 and CCL3 (Fig. 2). In addition, the ROS level of OGD-treated microglial cells significantly increased (Additional file 6: Fig. S3).

### LOX-1 knockdown shifted OGD-treated microglial cells to an anti-inflammatory phenotype

To investigate the relationship between LOX-1 and microglial activation, we analyzed whether microglial cells were activated under hypoxic and ischemic conditions and whether their activation was changed with LOX-1 siRNA administration. LOX-1 siRNA efficiently knocked down LOX-1 expression in microglial cells, and we confirmed that the expression of LOX-1 (*Olr1*) induced by OGD was suppressed by LOX-1 siRNA to the same level as that of the control (Additional file 5: Fig. S2). The production of the cytokines IL-1β, IL-6 and TNF-α was increased after OGD and suppressed by LOX-1 siRNA administration (Fig. 2A). Moreover, the production of the chemokines CCL2, CCL5 and CCL3 was increased after OGD and suppressed by LOX-1 siRNA administration (Fig. 2B). The expression level of *IL-4*, an anti-inflammatory cytokine, was not significantly changed (Additional file 7: Fig. S4). However, *Nos2* (iNOS) expression in OGD-treated microglial cells was increased compared with that of the control and was suppressed by LOX-1 siRNA (Additional file 8: Fig. S5). The ROS level in OGD-treated microglial cells was decreased to that of untreated microglial cells by LOX-1 siRNA treatment (Additional file 6: Fig. S3). These data clearly indicate that hypoxic/ischemic conditions induce the expression levels of inflammatory cytokines/chemokines, iNOS and ROS in microglial cells through the upregulation of LOX-1, and the downregulation of LOX-1 recovered the expression levels of these inflammatory mediators.

To further investigate the role of LOX-1 in microglial cells, we performed transcriptome analysis to compare the expression patterns in control (CTL)-treated, OGD-treated and OGD-treated LOX-1 siRNA-treated microglial cells. The heatmap indicates that LOX-1 knockdown shifted OGD-treated microglia to the M2 phenotype (Additional file 9: Fig. S6).

### LOX-1 inhibition altered the NF-κB distribution pattern and the phosphorylation of p38-MAPK

**Fig. 3:**
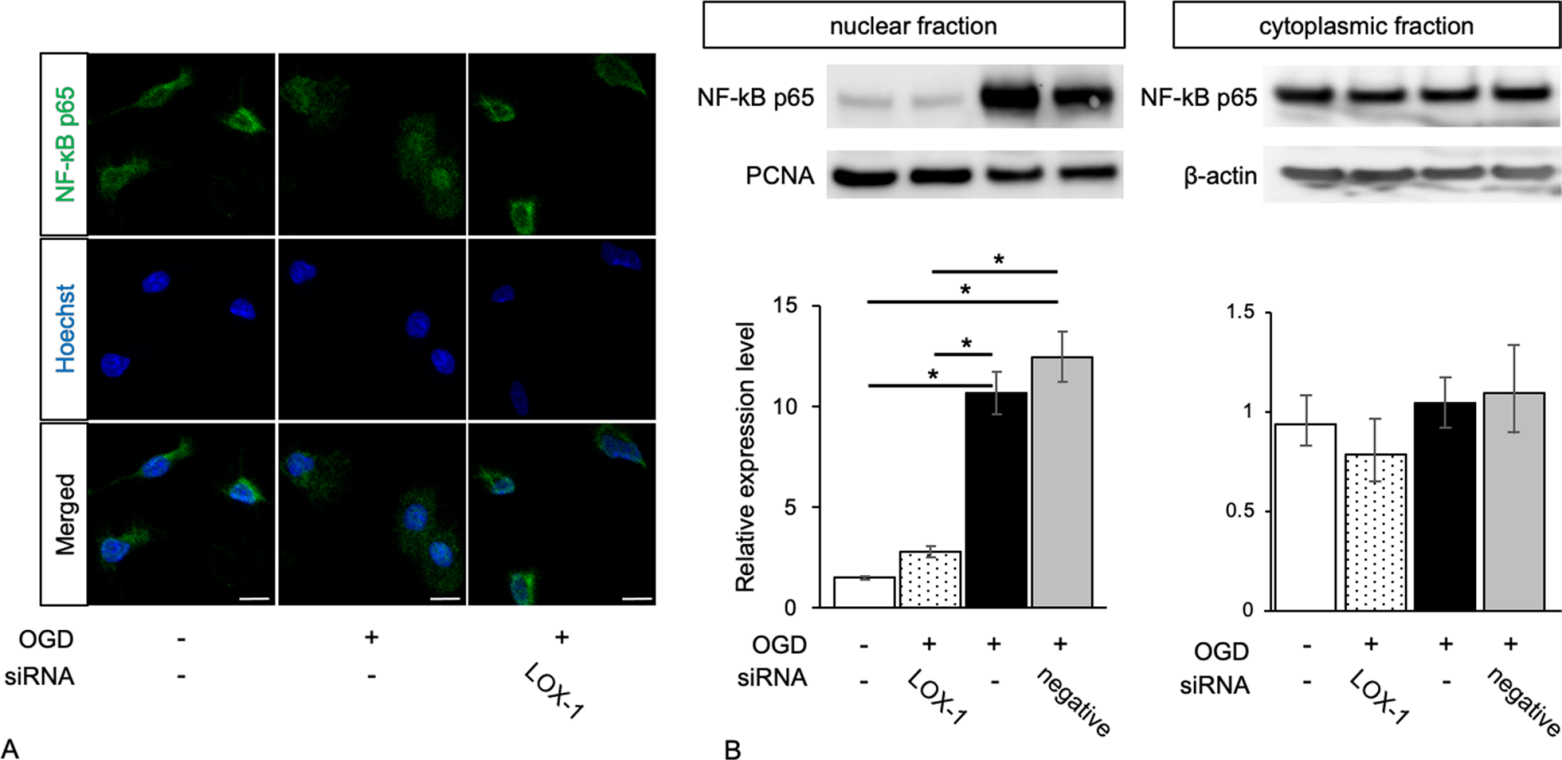
LOX-1 inhibition alters the distribution of NF-κB p65 from the cytoplasm to the nucleus. OGD-treated microglial cells show NF-κB p65 in the nucleus, but LOX-1 siRNA induces cytoplasmic expression of NF-κB p65 (**A**). Western blots exhibit significantly increased NF-κB p65 expression levels in the nuclear fraction of OGD-treated microglial cells but no significant difference in NF-κB p65 expression in the cytoplasmic fraction (**B**). Hoechst; Hoechst 33342, *P < 0.05. Scale bar = 10 µm

**Fig. 4:**
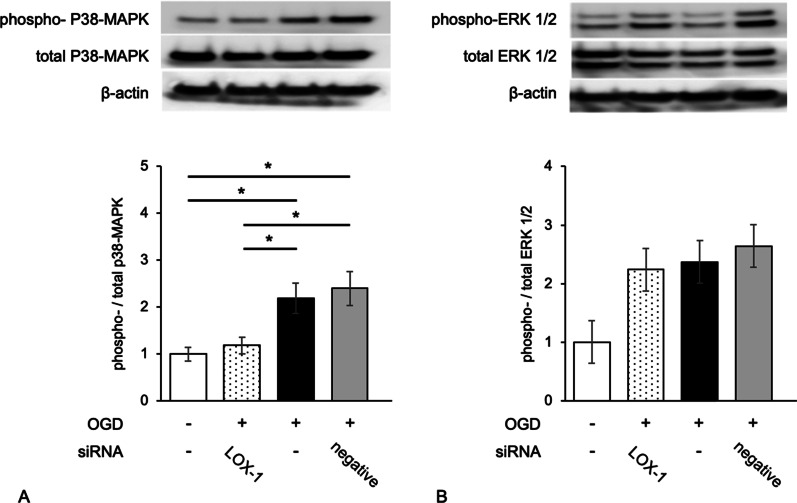
LOX-1 suppression induces different expression patterns of p38-MAPK and ERK phosphorylation in OGD-treated microglial cells. The expression of phospho-p38-MAPK and the ratio of phospho/total p38-MAPK were significantly increased in OGD-treated microglial cells but were reduced to the control level by LOX-1 siRNA (**A**). However, the expression of phospho-ERK and the ratio of phospho/total ERK were relatively increased in OGD-treated microglial cells but were not significantly reduced to the control level by LOX-1 siRNA (**B**). *P < 0.05

NF-κB p65 expression was examined in the cytoplasm of primary microglial cells (Fig. 3). After OGD induction, NF-κB p65 expression was mainly in the nucleus. However, LOX-1 knockdown in OGD-treated microglial cells recovered NF-κB p65 expression in the cytoplasm. As the intracellular signaling pathway of LOX-1 is known, we investigated p38-MAPK and ERK 1/2 activity and phosphorylation. OGD insult induced phosphorylation of p38-MAPK and ERK 1/2 (Fig. 4). LOX-1 siRNA treatment in OGD-treated microglial cells recovered the p38-MAPK phosphorylation level but not the ERK 1/2 phosphorylation level. These results indicated that p38-MAPK activation by OGD was dependent of the LOX-1 pathway.

### Effects of p38-MAPK and NF-κB inhibitors in OGD-treated microglial cells

**Fig. 5:**
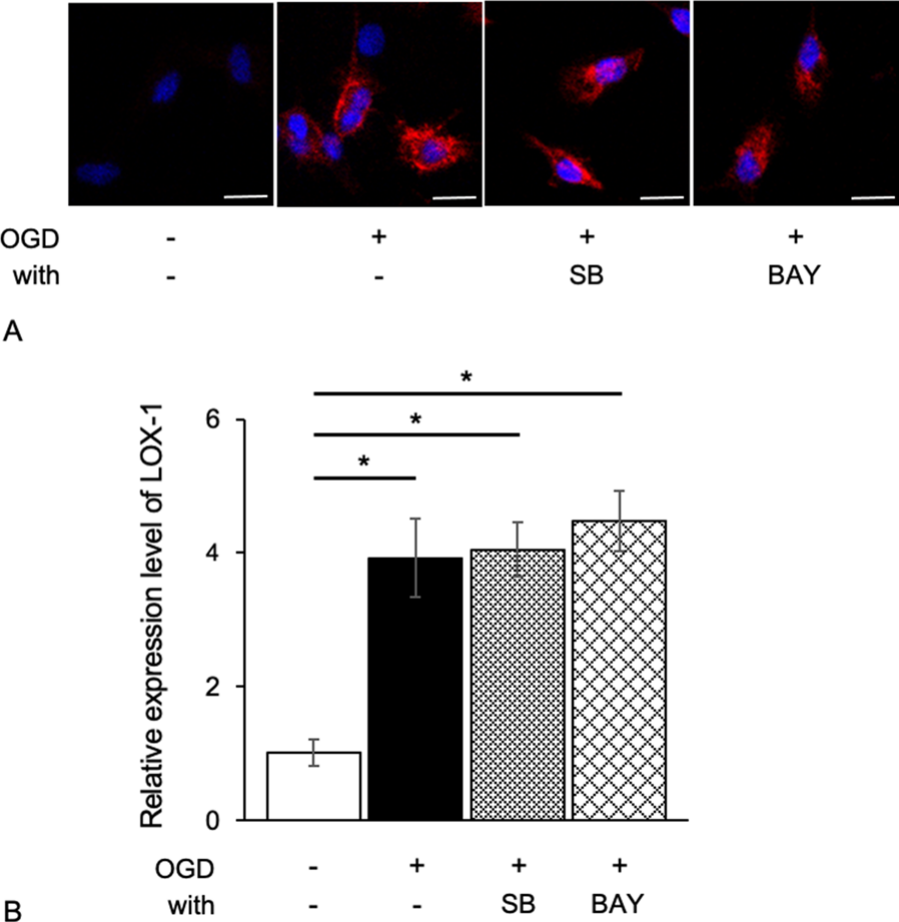
p38-MAPK and NF-κB inhibitors do not change LOX-1 expression levels in OGD-treated microglial cells. OGD-treated microglial cells expressed LOX-1 protein (red) in the cytoplasm (**A**). The same photographs of untreated microglial cells and OGD-treated microglial cells are shown in Fig. 1A, B. LOX-1 expression is not influenced by the p38-MAPK or NF-κB inhibitors SB or BAY, respectively. The relative LOX-1 expression level was the same as that shown by immunocytochemistry (**B**). SB; SB203580, BAY; BAY11-7082, Scale bar = 10 µm. *P < 0.05

**Fig. 6:**
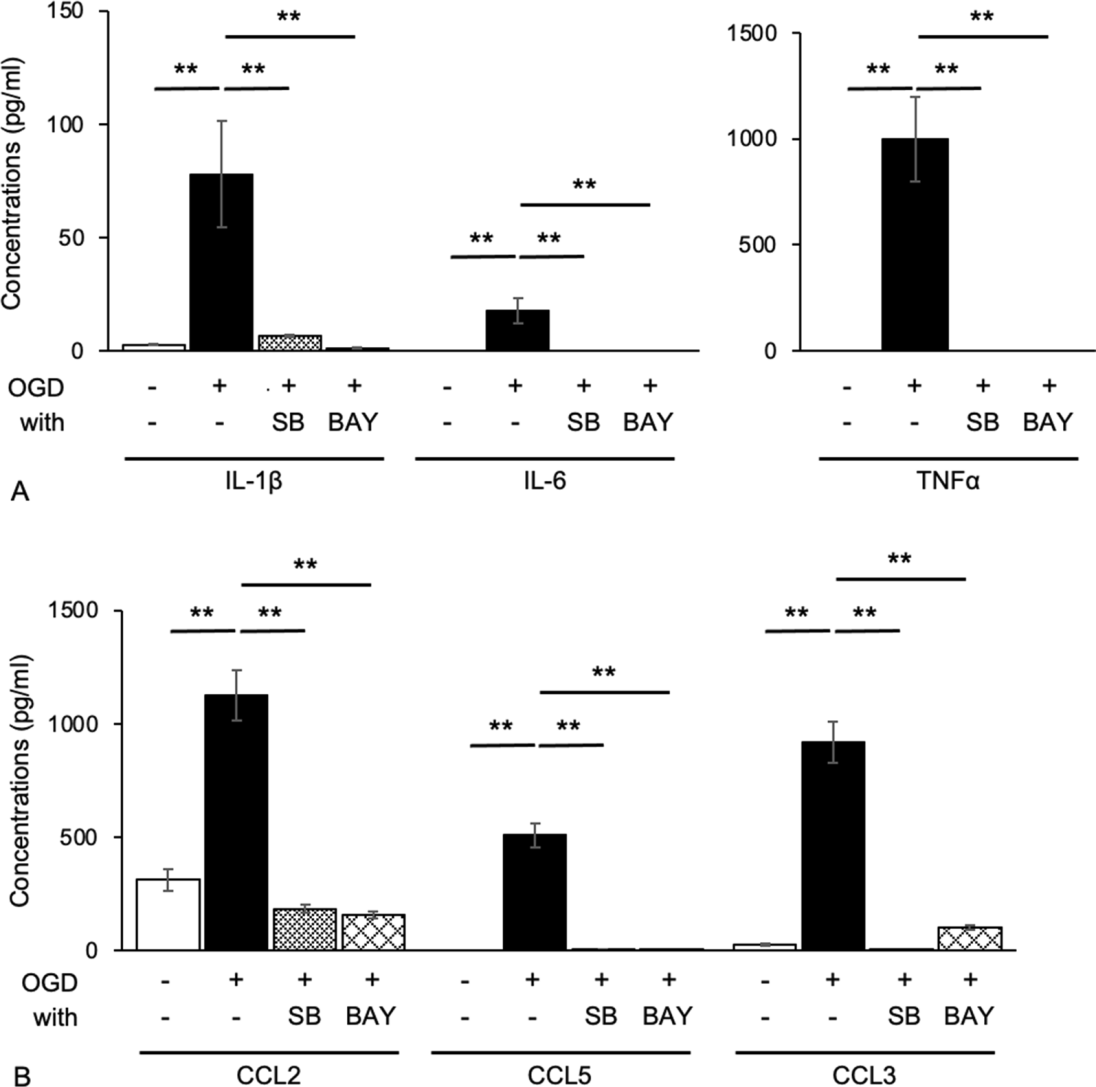
The production of cytokines and chemokines in OGD-treated microglial cells is downregulated by p38-MAPK and NF-κB inhibitors. The production of the cytokines IL-1β, IL-6 and TNF-α (**A**) and the chemokines CCL2, CCL5 and CCL3 (**B**), which are increased by OGD treatment, are suppressed by SB and BAY to the same levels as those of the control. SB; SB203580, BAY; BAY11-7082, **P < 0.01

SB203580 (SB), a p38-MAPK inhibitor, and BAY11-7082 (BAY), an NF-κB inhibitor, did not alter the LOX-1 expression pattern or levels in the cultured cells (Fig. 5). However, both SB and BAY markedly reduced the expression levels of the cytokines IL-1β, IL-6 and TNF-α and the chemokines CCL2, CCL5 and CCL3 (Fig. 6). These data indicate that NF-κB and p38-MAPK are transcription factors associated with cytokines and chemokines. Furthermore, LOX-1 is one of the OGD-induced cytokine/chemokine pathways in microglial cells, and NF-κB and p-38 MAPK are important components of these pathways.

**Fig. 7:**
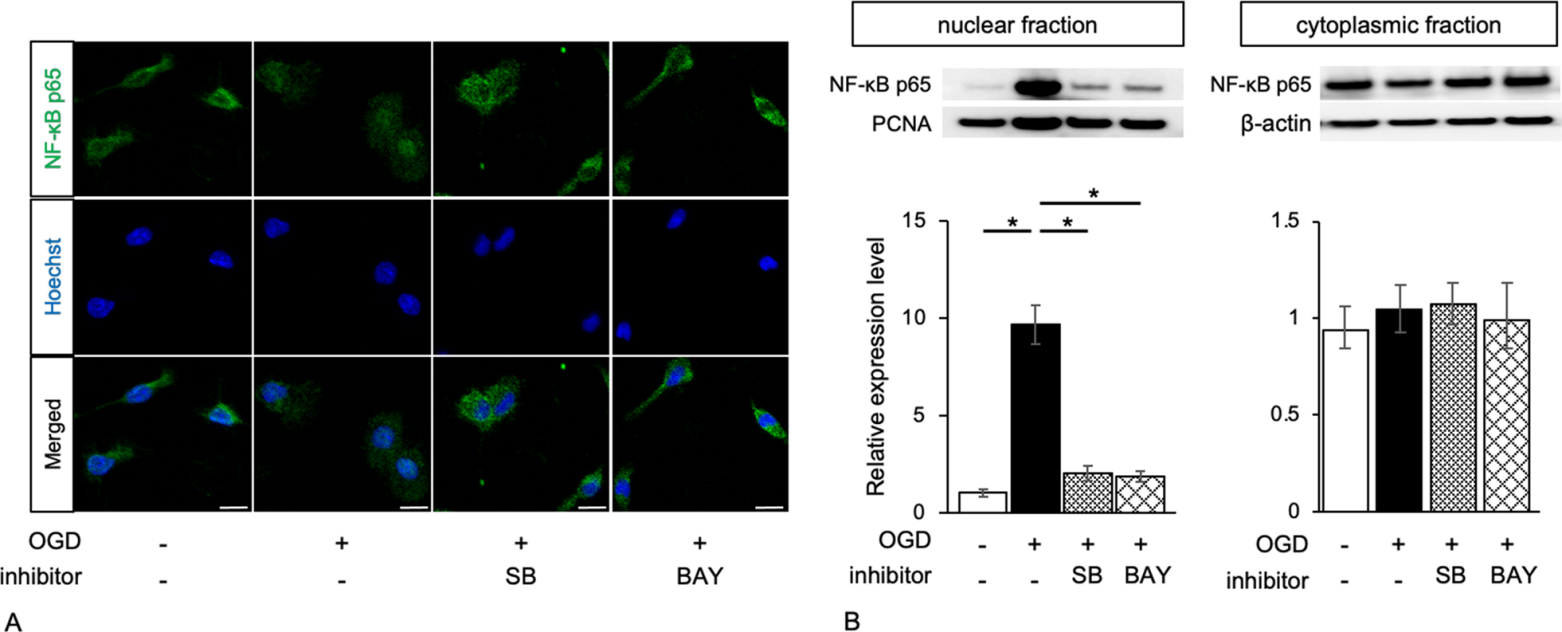
p38-MAPK and NF-κB inhibitors alter the distribution pattern of NF-κB p65 from the cytoplasm to the nucleus. NF-κB p65 is located in the nucleus in OGD-treated microglial cells but in the cytoplasm in other microglial cells (**A**). The same photographs of untreated microglial cells and OGD-treated microglial cells are shown in Fig. 3A. Western blots exhibit significantly increased NF-κB p65 expression levels in the nuclear fraction of OGD-treated microglial cells but no significant differences in NF-κB p65 expression levels in the cytoplasmic fraction (**B**). Hoechst; Hoechst 33,342, Scale bar = 10 µm. *P < 0.05

NF-κB expression was analyzed, and both inhibitors suppressed NF-κB p65 nuclear expression (Fig. 7). Furthermore, the ratio of phospho-p38-MAPK to total p38-MAPK (phospho/total p38-MAPK) was increased in OGD-treated microglia (Additional file 10: Fig. S7). SB reduced the ratio to the baseline level, but BAY did not have this effect. NF-κB may be downstream of p-38 MAPK.

Furthermore, we found that innate immune signaling pathways were significantly upregulated after OGD induction and that these upregulations were altered by LOX-1 siRNA, as determined by the gene set analysis. These findings suggested that the genes in the innate immune pathways may be downstream of LOX-1, and the upregulation of LOX-1 leads to microglial activation through these genes.

### Microglial cell viability

Cell viability were measured in in vitro experiments. Cell viability was not significantly changed after OGD compared with that of the control group (Additional file 11: Fig. S8).

### NF-κB and HIF-1 alpha induce OLR-1 promoter activity under the OGD conditions

**Fig. 8:**
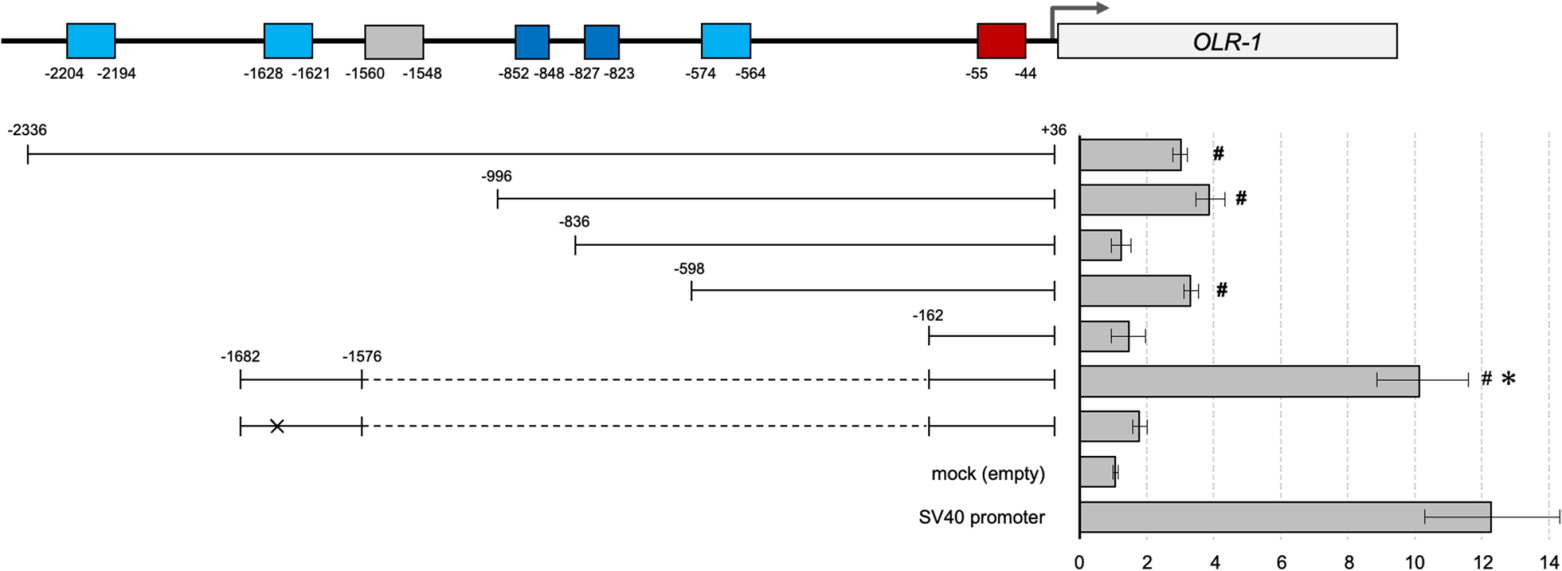
A luciferase reporter assay revealed the NF-κB binding site of − 1628 to − 1621 of the *OLR-1* promoter region. The *OLR-1* transcription activity is less affected by the binding sites of OCT-1 and HIF-1α. #*P* < 0.05 compared with the mock;  , not significant compared with the SV40 promoter.  , TATA box;  , NF-κB binding site;  , OCT-1 binding site;  , HIF-1α binding site;  , mutation position

To investigate whether a transcriptional binding site is present in the promoter of the *OLR-1* gene, a 2.3-kb upstream region of the promoter was cloned into the pGL3-luciferase reporter vector. This region contains three predicted putative transcription factor-binding sites, NF-κB, OCT-1 and HIF-1α. We used HMC-3 cells (a human microglial cell line) to conduct this promoter assay. As shown in Fig. 8, the region between − 1576 and − 1682 bp yielded the most substantial change in luciferase activity. Whereas NF-κB and HIF-1α are both transcriptional activators, NF-κB has strong transcriptional activity. However, OCT-1 is a transcriptional repressor but can be upregulated in the oxLDL-mediated LOX-1 pathway [36, 37]. However, OCT-1 may have minimum activity of *OLR-1* gene transcription.

Using the chromatin immunoprecipitation (ChIP) assay, we found that NF-κB and HIF-1α bind to the promoter region of the *OLR-1* gene (Additional file 12: Fig. S9).

## Discussion

In the present study, we found that LOX-1 was induced in microglial cells under oxygen and nutrient deprivation and mediated the inflammatory activation of microglial cells, which led to the production of inflammatory mediators such as cytokines, chemokines, and reactive oxygen/nitrogen species. Blocking the LOX-1 signal transduction pathway suppressed the production of inflammatory mediators. The OGD method is widely used to analyze the pathology of neurons, astrocytes, or myocytes as models of hypoxic and ischemic conditions [45–48]. We demonstrated that OGD-induced stress increased microglial cell expression of inflammatory cytokines (IL-1β, IL-6 and TNFα) and chemokines (CCL2, CCL5 and CCL3) (Fig. 2, Additional file 6: Fig. S3). Transcriptome analysis showed that OGD-induced microglial cells expressed inflammatory gene transcripts. In addition, the expression of LOX-1 was induced in OGD-treated microglial cells, which was similar to the phenomenon of microglial cells in the nHIE rat model [31]. However, LOX-1 knockdown in OGD-treated microglial cells led to the suppression of inflammatory mediators, which was consistent with the change observed in the nHIE rat model brain treated with an anti-LOX-1 neutralizing antibody [26]. Therefore, our OGD microglial cell model is a useful method to develop or screen drugs for HIE and inflammatory conditions in the brain and to analyze the LOX-1 signal transduction pathway as a treatment target. This finding also indicates that microglial cells have an LOX-1-inducible immune system, which acts under hypoxic/ischemic conditions.

LOX-1 expression is induced by external stimuli, such as ox-LDL, apoptotic cells, and cytokines [30, 49]. A study reported that conditioned medium derived from necrotic neuronal cultures induced LOX-1 expression in microglial cells [50]. HSP60 has also been identified as a molecule that induces LOX-1 expression, and HSP60 acts on LOX-1 expressing microglial cells to induce the production of inflammatory mediators [50]. However, we demonstrated that the expression of LOX-1 was induced in primary rat microglial cells after OGD treatment without any external stimulus. In addition, the upregulation of LOX-1 in OGD-treated microglial cells continued despite the suppression of inflammatory cytokines and chemokines with p38-MAPK or NF-κB inhibitors. This finding means that the induction of LOX-1 in microglial cells is not related to inflammatory cytokines or chemokines. Based on these results, we can speculate that the expression of LOX-1 is induced by not only external stimuli but also endogenous factors in microglial cells under hypoxic and ischemic conditions [51, 52].

Based on the results of the human *OLR1* promoter sequence and transcription factor-binding site prediction, we confirmed that HIF-1α and NF-κB can directly control LOX-1 expression. Under conditions of oxygen and nutrient deficiency, HIF-1α may first act on the immune system via microglial cells. However, NF-κB mainly acts as a transcription factor of the *OLR-1* gene. In addition, we can speculate that NF-κB induced by the cellular LOX-1 pathway leads to cytokine production. Our results revealed that NF-κB expression was induced by OGD insult and was localized in the nucleus (Fig. 3). This may demonstrate that LOX-1 is overexpressed in microglial cells by positive feedback of the LOX-1 pathway.

Our previous study demonstrated that nHIE model rats expressed LOX-1 and were cured by an anti-LOX-1 neutralizing antibody [26]. In this study, we showed that LOX-1 siRNA downregulated 60–80% of LOX-1 mRNA expression and protein expression to the baseline level (Additional file 5: Fig. S2). Namely, defects in the brain circulatory system lead to LOX-1 expression, and suppression of LOX-1 can markedly alleviate this pathology. Thus, LOX-1 is considered a major candidate for nHIE therapy. It is known to be an important role in the activity of p38-MAPK and NF-κB in the LOX-1 intracellular signaling pathway [30]. Therefore, we investigated the activation of the both key molecules under the OGD condition. OGD insult induced P38-MAPK activation but did not influence ERK1/2 activation; however, both molecules are components of the cellular LOX-1 pathway (Fig. 4). Moreover, inhibitors of P38-MAPK and NF-κB reduced cytokine and chemokine expression to levels similar to those of LOX-1 siRNA (Fig. 6). However, the inhibitors may affect the other cellular signaling pathways.

As we previously described, activated microglial cells produce inflammatory cytokines and chemokines and reactive oxygen/nitrogen species under hypoxic and ischemic conditions. Inflammatory cytokines and chemokines activate immune cells, including microglial cells, and induce migration of these immune cells and destruction of the blood–brain barrier [53]. Therefore, these cytokines and chemokines may indirectly cause neuronal injuries. In contrast, neurons express TNFα receptors and TNFα can directly induce apoptosis via caspase-8 activation or necrosis RIP1 and RIP3 activation in neurons [53, 54]. Neurons also express IL-1β receptors, and hypoxic microglia-derived IL-1β induces neuronal apoptosis via these receptors [55]. Therefore, in addition to indirect neuronal injury, some cytokines can directly cause neuronal injury. In this study, the production of these cytokines was related to the expression of LOX-1 in hypoxic and ischemic-injured microglial cells, which means that the upregulation of LOX-1 in activated microglial cells is indirectly or directly related to neuronal injuries.

Recently, it has been shown that microglial cells contribute to the pathophysiology of various neurodegenerative diseases, epilepsy, neurodevelopmental diseases, autoimmune encephalitis, neurologic pain and brain trauma [56]. Hypoxic and ischemic brain injuries are caused by many circulatory dysfunctions, extrinsic accidents and various pathological conditions. nHIE is a major perinatal disease and results in lifelong neurological and mental deficiency. A completely curative therapy is needed. LOX-1 and its related molecules or chemicals are major candidates for nHIE therapy.

## Conclusion

We investigated the alteration in LOX-1 expression in microglia and the influence of LOX-1 on detrimental processes under hypoxic/ischemic conditions using primary rat microglial cell cultures. We performed oxygen–glucose deprivation (OGD) treatment as an in vitro model of hypoxia/ischemia and LOX-1 knockdown using siRNA and treated the cells with the inhibitors of p38-MAPK or NF-κB. OGD-treated microglial cells induced LOX-1, cytokine and chemokine expression and ROS production. This finding indicates that hypoxic/ischemic conditions of microglial cells induced LOX-1 expression and activated the immune system through NF-κB activation. Moreover, LOX-1 reduction with LOX-1 siRNA shifted microglial cells to an anti-inflammatory phenotype and reduced the expression levels of cytokines and chemokines to levels similar to those of P38-MAPK or NF-κB inhibitors. Here, we revealed one of the LOX-1 signaling pathways in microglia and suggest a possible a new treatment strategy for nHIE.

## Supplementary Information

**Additional file 1: Table S1**. Primer sequences for luciferase reporter assay

**Additional file 2: Table S1**. Primer sequences for luciferase reporter assay

**Additional file 3: Table S2**. Primer sequences for chromotin immunoprecipitation assay

**Additional file 4: Figure S1**. Primary microglial cells derived from premature rat brains were confirmed by Iba-1 immunocytochemistry. We obtained more than 98% of Iba-1 positive microglia. Scale bar = 50 μm.

**Additional file 5: Figure S2**. The efficacy of LOX-1 siRNA was evaluated by quantitative PCR and Western blot analyses. At 24 h after LOX-1 siRNA treatment, LOX-1 gene expression levels were downregulated by approximately 75% in microglial cells without siRNA treatment. LOX-1 protein expression was reduced to the same level as that in the control. *P < 0.05.

**Additional file 6: Figure S3**. LOX-1 siRNA suppresses ROS production in OGD-treated microglial cells. OGD-treated microglial cells exhibit significant oxidative stress, showing positivity for CellRox Green Reagent. The relative intensity per cell of OGD-treated microglial cells was approximately 20 times that of the control. NAC; *N*-acetyl cysteine. Scale bar = 20 μm. **P < 0.01.

**Additional file 7: Figure S4**. LOX-1 siRNA suppresses IL-4 in OGD-treated microglial cells. OGD-treated microglial cells exhibit significant IL-4 concentration. *P < 0.05.

**Additional file 8: Figure S5**. OGD induces Nos2 expression. LOX-1 siRNA suppresses Nos2 expression in OGD-treated microglial cells. **P < 0.01.

**Additional file 9: Figure S6**. LOX-1 suppression in OGD-treated microglial cells changes the inflammation-related gene expression pattern. Primary microglial cells express anti-inflammatory gene transcripts. In contrast, OGD-treated microglial cells expressed inflammatory gene transcripts. However, LOX-1 siRNA shifts the M2-transcription pattern.

**Additional file 10: Figure S7**. p38-MAPK and NF-κB inhibitors show different expression patterns of phosphorylated p38-MAPK in OGD-treated microglial cells. The ratio of phospho/total p38-MAPK was significantly higher level in OGD-treated microglial cells, but was reduced to the control level with SB. However, BAY did not reduce the ratio of phospho/total p38-MAPK. SB; SB203580, BAY; BAY11-7082, *P < 0.05, **P < 0.01.

**Additional file 11: Figure S8**. Microglial cell viability is not affected by OGD. There are no significant.

**Additional file 12: Figure S9**. The chromatin immunoprecipitation assay demonstrated that the both transcription factors of NF-κB and HIF-1α bind to the *OLR-1* gene promoter region under the OGD conditions. M; 100-bp ladder marker, 1; I-κBa promoter region as a control, 2; VEGFA promoter region as a control, 3; ③ of NF-κB binding site, 4; ④ of HIF-1α binding site, 5; ⑤ of HIF-1α binding site, 6; ⑥ of NF-κB binding site, D; distilled water.

## Data Availability

All data are contained within the article and supporting information. The datasets and analyzed data during this study are available from the corresponding author on reasonable request with permission from the Ethics Committee of the National Center of Neurology and Psychiatry.

## Abbreviations

CCL2: C-C motif chemokine 2
CCL3: C-C motif chemokine 3
CCL5: C-C motif chemokine 5
CNS: Central nervous system
CTL: Control
DMEM: Dulbecco’s modified Eagle’s medium
FBS: Fetal bovine serum
HIF-1α: Hypoxia inducible Factor 1α
IL-1β: Interleukin-1β
IL-6: Interleukin-6
LOX-1: Lectin-like oxidized low-density lipoprotein receptor-1
OCT-1: Octamer-binding transcription Factor 1
OGD: Oxygen glucose deprivation
OLR1: Oxidized low-density lipoprotein receptor 1
oxLDL: Oxidized low-density lipoprotein
NAC: *N*-acetyl cysteine
NF-κB: Nuclear factor-kappa B
nHIE: Neonatal hypoxic-ischemic encephalopathy
p38-MAPK: P-38 mitogen-activated protein kinase
PFA: Paraformaldehyde
ROS: Reactive oxygen species
TNF-α: Tumor necrosis factor-α

## References

[1] Ginhoux F, Greter M, Leboeuf M, Nandi S, See P, Gokhan S (2010). Fate mapping analysis reveals that adult microglia derive from primitive macrophages. Science.

[2] Saijo K, Glass CK (2011). Microglial cell origin and phenotypes in health and disease. Nat Rev Immunol.

[3] Schulz C, Gomez Perdiguero E, Chorro L, Szabo-Rogers H, Cagnard N, Kierdorf K (2012). A lineage of myeloid cells independent of Myb and hematopoietic stem cells. Science.

[4] Faustino JV, Wang X, Johnson CE, Klibanov A, Derugin N, Wendland MF (2011). Microglial cells contribute to endogenous brain defenses after acute neonatal focal stroke. J Neurosci.

[5] Jin WN, Shi SX, Li Z, Li M, Wood K, Gonzales RJ (2017). Depletion of microglia exacerbates postischemic inflammation and brain injury. J Cereb Blood Flow Metab.

[6] Bhalala US, Koehler RC, Kannan S (2015). Neuroinflammation and neuroimmune dysregulation after acute hypoxic-ischemic injury of developing brain. Front Pediatr.

[7] Dommergues MA, Plaisant F, Verney C, Gressens P (2003). Early microglial activation following neonatal excitotoxic brain damage in mice: a potential target for neuroprotection. Neuroscience.

[8] Hu X, Leak RK, Shi Y, Suenaga J, Gao Y, Zheng P (2015). Microglial and macrophage polarization—new prospects for brain repair. Nat Rev Neurol.

[9] Lan X, Han X, Li Q, Yang QW, Wang J (2017). Modulators of microglial activation and polarization after intracerebral haemorrhage. Nat Rev Neurol.

[10] Orihuela R, McPherson CA, Harry GJ (2016). Microglial M1/M2 polarization and metabolic states. Br J Pharmacol.

[11] Fumagalli M, Lombardi M, Gressens P, Verderio C (2018). How to reprogram microglia toward beneficial functions. Glia.

[12] Jaworska J, Ziemka-Nalecz M, Sypecka J, Zalewska T (2017). The potential neuroprotective role of a histone deacetylase inhibitor, sodium butyrate, after neonatal hypoxia-ischemia. J Neuroinflamm.

[13] Kanazawa M, Miura M, Toriyabe M, Koyama M, Hatakeyama M, Ishikawa M (2017). Microglia preconditioned by oxygen-glucose deprivation promote functional recovery in ischemic rats. Sci Rep.

[14] Truettner JS, Bramlett HM, Dietrich WD (2017). Posttraumatic therapeutic hypothermia alters microglial and macrophage polarization toward a beneficial phenotype. J Cereb Blood Flow Metab.

[15] Fleiss B, Van Steenwinckel J, Bokobza C, Shearer IK, Ross-Munro E, Gressens P (2021). Microglia-mediated neurodegeneration in perinatal brain injuries. Biomolecules.

[16] Volpe JJ (2019). Dysmaturation of premature brain: importance, cellular mechanisms, and potential interventions. Pediatr Neurol.

[17] Kaur C, Rathnasamy G, Ling EA (2013). Roles of activated microglia in hypoxia induced neuroinflammation in the developing brain and the retina. J Neuroimmune Pharmacol.

[18] Baburamani AA, Supramaniam VG, Hagberg H, Mallard C (2014). Microglia toxicity in preterm brain injury. Reprod Toxicol.

[19] Back SA (2017). White matter injury in the preterm infant: pathology and mechanisms. Acta Neuropathol.

[20] Zaghloul N, Kurepa D, Bader MY, Nagy N, Ahmed MN (2020). Prophylactic inhibition of NF-κB expression in microglia leads to attenuation of hypoxic ischemic injury of the immature brain. J Neuroinflamm.

[21] Douglas-Escobar M, Weiss MD (2015). Hypoxic-ischemic encephalopathy: a review for the clinician. JAMA Pediatr.

[22] Volpe JJ (2012). Neonatal encephalopathy: an inadequate term for hypoxic-ischemic encephalopathy. Ann Neurol.

[23] Kurinczuk JJ, White-Koning M, Badawi N (2010). Epidemiology of neonatal encephalopathy and hypoxic-ischaemic encephalopathy. Early Hum Dev.

[24] van Bel F, Groenendaal F (2016). Drugs for neuroprotection after birth asphyxia: pharmacologic adjuncts to hypothermia. Semin Perinatol.

[25] Jacobs SE, Berg M, Hunt R, Tarnow-Mordi WO, Inder TE, Davis PG (2013). Cooling for newborns with hypoxic ischaemic encephalopathy. Cochrane Database Syst Rev.

[26] Akamatsu T, Dai H, Mizuguchi M, Goto Y, Oka A, Itoh M (2014). LOX-1 is a novel therapeutic target in neonatal hypoxic-ischemic encephalopathy. Am J Pathol.

[27] Sawamura T, Kume N, Aoyama T, Moriwaki H, Hoshikawa H, Aiba Y (1997). An endothelial receptor for oxidized low-density lipoprotein. Nature.

[28] Brown GD, Willment JA, Whitehead L (2018). C-type lectins in immunity and homeostasis. Nat Rev Immunol.

[29] De Siqueira J, Abdul Zani I, Russell DA, Wheatcroft SB, Ponnambalam S, Homer-Vanniasinkam S (2015). Clinical and preclinical use of LOX-1-specific antibodies in diagnostics and therapeutics. J Cardiovasc Transl Res.

[30] Taye A, El-Sheikh AA (2013). Lectin-like oxidized low-density lipoprotein receptor 1 pathways. Eur J Clin Invest.

[31] Akamatsu T, Sugiyama T, Oshima T, Mizukami A, Goishi K, Shichino H (2021). Lectin-like oxidized low-density lipoprotein receptor-1 related microglial activation and proliferation in neonatal hypoxic-ischemic encephalopathy: microglial morphology considerations. Am J Pathol.

[32] Chalak LF, Sánchez PJ, Adams-Huet B, Laptook AR, Heyne RJ, Rosenfeld CR (2014). Biomarkers for severity of neonatal hypoxic-ischemic encephalopathy and outcomes in newborns receiving hypothermia therapy. J Pediatr.

[33] Liu F, McCullough LD (2013). Inflammatory responses in hypoxic ischemic encephalopathy. Acta Pharmacol Sin.

[34] Silveira RC, Procianoy RS (2003). Interleukin-6 and tumor necrosis factor-alpha levels in plasma and cerebrospinal fluid of term newborn infants with hypoxic-ischemic encephalopathy. J Pediatr.

[35] Hermonat PL, Zhu H, Cao M, Mehta JL (2011). LOX-1 transcription. Cardiovasc Drugs Ther.

[36] Chen J, Liu Y, Liu H, Hermonat PL, Mehta JL (2006). Lectin-like oxidized low-density lipoprotein receptor-1 (LOX-1) transcriptional regulation by Oct-1 in human endothelial cells: implications for atherosclerosis. Biochem J.

[37] Thum T, Borlak J (2008). LOX-1 receptor blockade abrogates oxLDL-induced oxidative DNA damage and prevents activation of the transcriptional repressor Oct-1 in human coronary arterial endothelium. J Biol Chem.

[38] Kröncke KD (2003). Nitrosative stress and transcription. Biol Chem.

[39] Bao L, Li RH, Li M, Jin MF, Li G, Han X (2017). Autophagy-regulated AMPAR subunit upregulation in in vitro oxygen glucose deprivation/reoxygenation-induced hippocampal injury. Brain Res.

[40] Gao Y, Wang Z, He W, Ma W, Ni X (2019). Mild hypothermia protects neurons against oxygen glucose deprivation via poly (ADP-ribose) signaling. J Matern Fetal Neonatal Med.

[41] Krech J, Tong G, Wowro S, Walker C, Rosenthal LM, Berger F (2017). Moderate therapeutic hypothermia induces multimodal protective effects in oxygen-glucose deprivation/reperfusion injured cardiomyocytes. Mitochondrion.

[42] Ou-Yang L, Liu Y, Wang BY, Cao P, Zhang JJ, Huang YY (2018). Carnosine suppresses oxygen-glucose deprivation/recovery-induced proliferation and migration of reactive astrocytes of rats in vitro. Acta Pharmacol Sin.

[43] Chen XP, Zhang TT, Du GH (2007). Lectin-like oxidized low-density lipoprotein receptor-1, a new promising target for the therapy of atherosclerosis?. Cardiovasc Drug Rev.

[44] Zhang D, Sun L, Zhu H, Wang L, Wu W, Xie J (2012). Microglial LOX-1 reacts with extracellular HSP60 to bridge neuroinflammation and neurotoxicity. Neurochem Int.

[45] Zhang W, Zhu T, Wu W, Ge X, Xiong X, Zhang Z (2018). LOX-1 mediated phenotypic switching of pulmonary arterial smooth muscle cells contributes to hypoxic pulmonary hypertension. Eur J Pharmacol.

[46] Zhu TT, Zhang WF, Luo P, Qian ZX, Li F, Zhang Z (2017). LOX-1 promotes right ventricular hypertrophy in hypoxia-exposed rats. Life Sci.

[47] Brown GC, Vilalta A (2015). How microglia kill neurons. Brain Res.

[48] Kraft AD, McPherson CA, Harry GJ (2009). Heterogeneity of microglia and TNF signaling as determinants for neuronal death or survival. Neurotoxicology.

[49] Kaur C, Sivakumar V, Zou Z, Ling EA (2014). Microglia-derived proinflammatory cytokines tumor necrosis factor-alpha and interleukin-1beta induce Purkinje neuronal apoptosis via their receptors in hypoxic neonatal rat brain. Brain Struct Funct.

[50] Salter MW, Stevens B (2017). Microglia emerge as central players in brain disease. Nat Med.

[51] Giulian D, Baker TJ (1986). Characterization of ameboid microglia isolated from developing mammalian brain. J Neurosci.

[52] Sanagi T, Yabe T, Yamada H (2005). The regulation of pro-inflammatory gene expression induced by pigment epithelium-derived factor in rat cultured microglial cells. Neurosci Lett.

[53] Hu YY, Wang Y, Liang S, Yu XL, Zhang L, Feng LY (2016). Senkyunolide I attenuates oxygen-glucose deprivation/reoxygenation-induced inflammation in microglial cells. Brain Res.

[54] Nakagomi T, Kubo S, Nakano-Doi A, Sakuma R, Lu S, Narita A (2015). Brain vascular pericytes following ischemia have multipotential stem cell activity to differentiate into neural and vascular lineage cells. Stem Cells.

[55] Yuan T, Li Z, Li X, Yu G, Wang N, Yang X (2014). Lidocaine attenuates lipopolysaccharide-induced inflammatory responses in microglia. J Surg Res.

[56] Yang L, Liu CC, Zheng H, Kanekiyo T, Atagi Y, Jia L (2016). LRP1 modulates the microglial immune response via regulation of JNK and NF-κB signaling pathways. J Neuroinflamm.

