# Supplementary material for: LOX-1 mediates inflammatory activation of microglial cells through the p38-MAPK/NF-κB pathways under hypoxic-ischemic conditions
Source: Cell Commun Signal. 2023 Jun 2;21:126. doi: 10.1186/s12964-023-01048-w (PMC10236821; doi:10.1186/s12964-023-01048-w)
Supplement: Supplementary file 2 — Additional file 1: Table S1. Primer sequences for luciferase reporter assay [file 12964_2023_1048_MOESM1_ESM.pdf]

Supplementary Table 1. Primer sequences for luciferase reporter assay

		(forward) primer sequence	reverse primer sequence
OLR-1 gene promoter	-1682 ~ Xho1*	ATCTG**CTCGAG**TCAGTGTGATATCGTTTCAG	
	-1682 ~ KpnI*	ATCTG**GGTACC**TCAGTGTGATATCGTTTCAG	
	-1455 ~ XhoI*	TAGCT**GAATTC**CTCATGCCTGTAATCTCAGC	
	-885 ~ Xho1*	ATCTG**CTCGAG**TGCTCCAAGTACAAAGCTTG	
	-885 ~ KpnI*	ATCTG**GGTACC**TGCTCCAAGTACAAAGCTTG	
	-847 ~ Xho1*	ATCTG**CTCGAG**TCATTTGAATTCCTATAGC	
	-847 ~ KpnI*	ATCTG**GGTACC**TCATTTGAATTCCTATAGC	
	-694 ~ Xho1*	ATCTG**CTCGAG**CAATGTTATCAGGTAGAC	
	-446 ~ XhoI*	ATCTG**CTCGAG**GACACCATATAGAAACAGTC	
	-446 ~ KpnI*	ATCTG**GAATTC**GACACCATATAGAAACAGTC	
	-162 ~ XhoI*	ATCTG**CTCGAG**TCCCAATATGAAGCAAAGCC	
	-107 ~ -1455	GATTACAGGCATGAGTCCCAATATGAAGCAAAGCCTCTCC	TGCTTCATATTGGGACTCATGCCTGTAATCTCA
	-1621 ~ - 1628 #	ACGCGTCGCTCTGTTGCCCAGGCTGGAGTAC	CCTTTAAAAAAAAAAAAAAAAAAGAATTAAAAAATACTG
pGL3-Basic vector	GL primer2	CTTTATGTTTTTGGCGTCTTCCA	
	RV primer3	CTAGCAAAATAGGCTGTCCC	

* ; pGL3-Basic vector cloning site, # ; the primer set for NF-κB binding site mutation. Bold sequences are the transcription binding sites.
