# Supplementary material for: LOX-1 mediates inflammatory activation of microglial cells through the p38-MAPK/NF-κB pathways under hypoxic-ischemic conditions
Source: Cell Commun Signal. 2023 Jun 2;21:126. doi: 10.1186/s12964-023-01048-w (PMC10236821; doi:10.1186/s12964-023-01048-w)
Supplement: Supplementary file 4 — Additional file 3: Table S2. Primer sequences for chromotin immunoprecipitation assay [file 12964_2023_1048_MOESM3_ESM.pdf]

Supplementary Table 2. Primer sequences for chromotin immunoprecipitation assay

	forward primer sequence	reverse primer sequence	product (bp)
Control			
IκBα gene promoter	GACGACCCCAATTCAAATCG	TCAGGCTCGGGGAATTTCC	300
VEGFA gene promoter	CCTCAGTTCCCTGGCAACATCTG	GAAGAATTTGGCACCAAGTTTGT	200
OLR-1 gene promoter			
-1734 ~ -1509	TTCATGGCATCCAAAATAACTG	GCCTGGTTGCAAGCCTATAA	226
-1005 ~ -835	AAGCGTCTTTGTGACATGAGTC	GAATTCAAATGACACGCTGGT	170
-829 ~ -640	GCAACGTGACGTTTTCACAA	TTTGAATCTATGTCTTTGTCTGGAA	191
-618 ~-425	GTAGACTAAAGGAAAGTACT	CACTGTTTTATCTGGCAATGGA	194
