# Supplementary material for: LOX-1 mediates inflammatory activation of microglial cells through the p38-MAPK/NF-κB pathways under hypoxic-ischemic conditions
Source: Cell Commun Signal. 2023 Jun 2;21:126. doi: 10.1186/s12964-023-01048-w (PMC10236821; doi:10.1186/s12964-023-01048-w)
Supplement: Supplementary file 5 — Additional file 4: Figure S1. Primary microglial cells derived from premature rat brains were confirmed by Iba-1 immunocytochemistry. We obtained more than 98% of Iba-1 positive microglia. Scale bar = 50 μm. [file 12964_2023_1048_MOESM4_ESM.pdf]

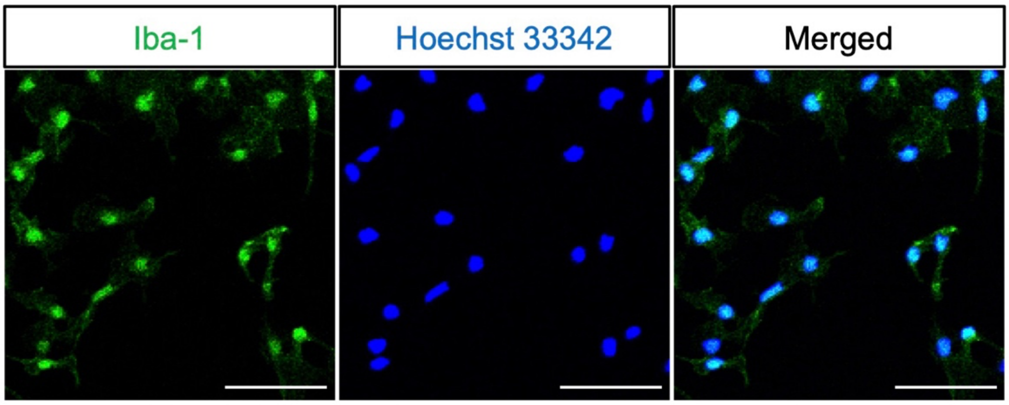

Supplementary Fig. 1. Primary microglial cells derived from premature rat brains were confirmed by Iba-1 immunocytochemistry. We obtained more than 98% of Iba-1 positive microglia. Scale bar = 50 μm.
