# Supplementary material for: LOX-1 mediates inflammatory activation of microglial cells through the p38-MAPK/NF-κB pathways under hypoxic-ischemic conditions
Source: Cell Commun Signal. 2023 Jun 2;21:126. doi: 10.1186/s12964-023-01048-w (PMC10236821; doi:10.1186/s12964-023-01048-w)
Supplement: Supplementary file 6 — Additional file 5: Figure S2. The efficacy of LOX-1 siRNA was evaluated by quantitative PCR and Western blot analyses. At 24 h after LOX-1 siRNA treatment, LOX-1 gene expression levels were downregulated by approximately 75% in microglial cells without siRNA treatment. LOX-1 protein expression was reduced to the same level as that in the control. *P < 0.05. [file 12964_2023_1048_MOESM5_ESM.pdf]

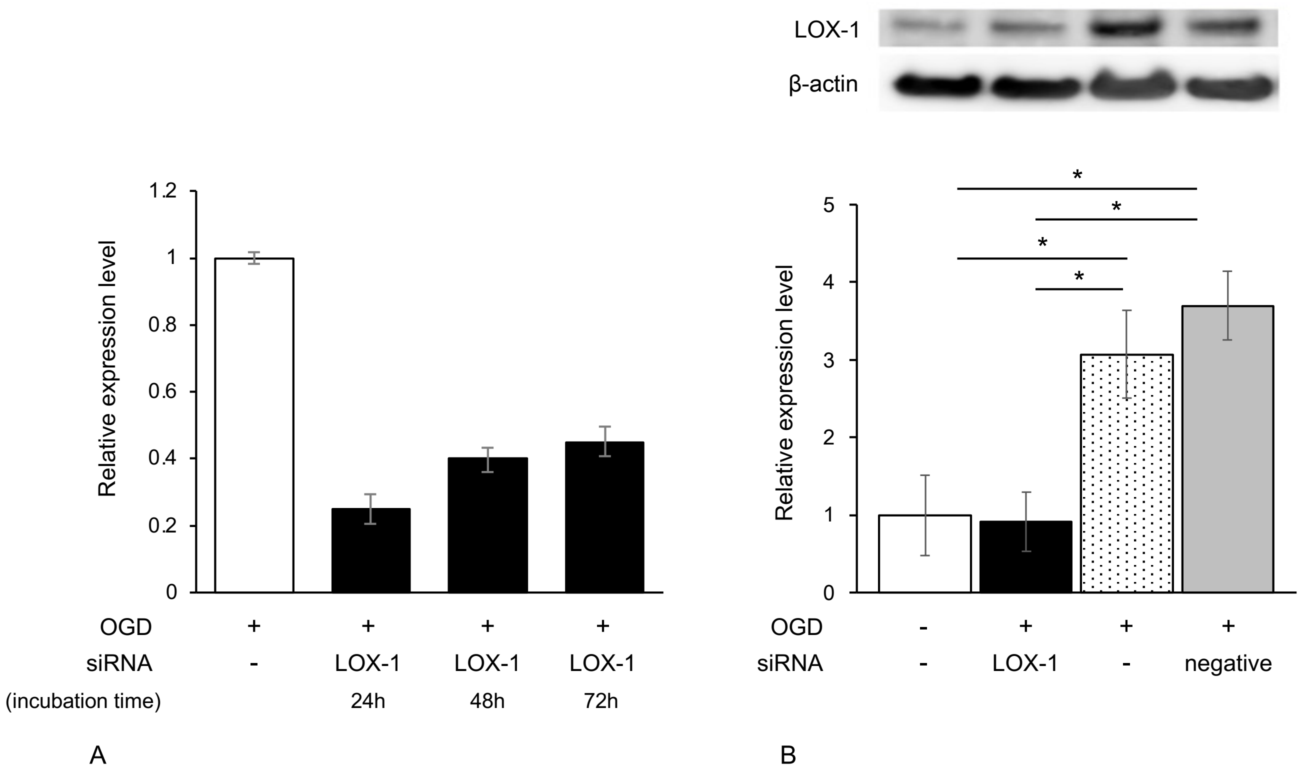

Supplementary Fig. 2. The efficacy of LOX-1 siRNA was evaluated by quantitative PCR and Western blot analyses. At 24 hours after LOX-1 siRNA treatment, LOX-1 gene expression levels were downregulated by approximately 75% in microglial cells without siRNA treatment (A) (N=6). LOX-1 protein expression was reduced to the same level as that in the control (B) (N=5). *: P<0.05.
