# Supplementary material for: LOX-1 mediates inflammatory activation of microglial cells through the p38-MAPK/NF-κB pathways under hypoxic-ischemic conditions
Source: Cell Commun Signal. 2023 Jun 2;21:126. doi: 10.1186/s12964-023-01048-w (PMC10236821; doi:10.1186/s12964-023-01048-w)
Supplement: Supplementary file 7 — Additional file 6: Figure S3. LOX-1 siRNA suppresses ROS production in OGD-treated microglial cells. OGD-treated microglial cells exhibit significant oxidative stress, showing positivity for CellRox Green Reagent. The relative intensity per cell of OGD-treated microglial cells was approximately 20 times that of the control. NAC; N-acetyl cysteine. Scale bar = 20 μm. **P < 0.01. [file 12964_2023_1048_MOESM6_ESM.pdf]

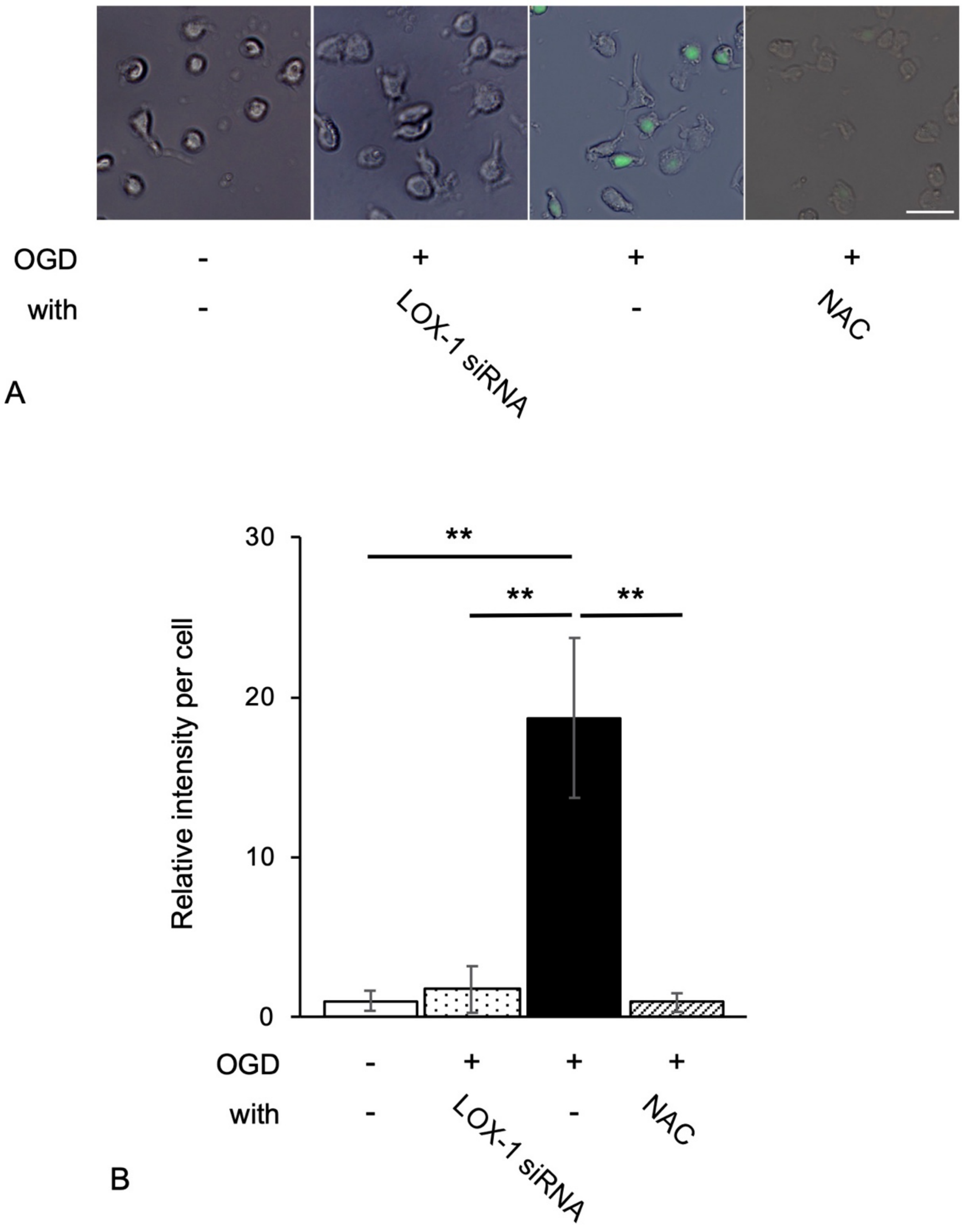

Supplementary Fig. 3. LOX-1 siRNA suppresses ROS production in OGD-treated microglial cells. OGD-treated microglial cells exhibit significant oxidative stress, showing positivity for CellRox Green Reagent (A). The relative intensity per cell of OGD-treated microglial cells was approximately 20 times that of the control (non-OGD treated microglial cells) (B). NAC; N-acetyl cysteine. Scale bar = 20 μm. **: $P<0.01$.
