# Supplementary material for: LOX-1 mediates inflammatory activation of microglial cells through the p38-MAPK/NF-κB pathways under hypoxic-ischemic conditions
Source: Cell Commun Signal. 2023 Jun 2;21:126. doi: 10.1186/s12964-023-01048-w (PMC10236821; doi:10.1186/s12964-023-01048-w)
Supplement: Supplementary file 8 — Additional file 7: Figure S4. LOX-1 siRNA suppresses IL-4 in OGD-treated microglial cells. OGD-treated microglial cells exhibit significant IL-4 concentration. *P < 0.05. [file 12964_2023_1048_MOESM7_ESM.pdf]

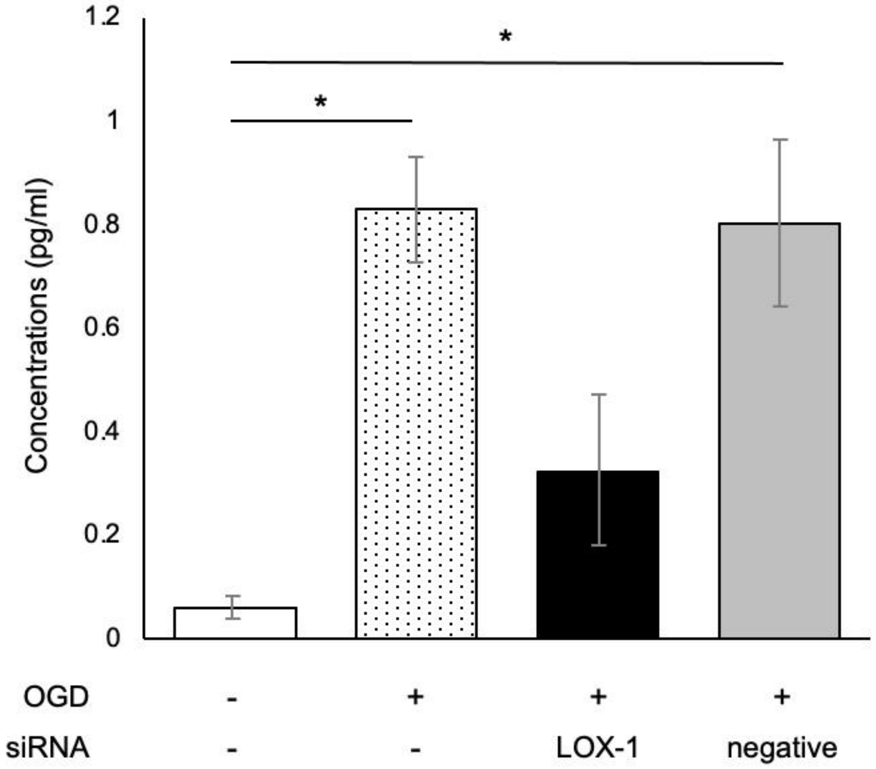

Supplementary Fig. 4. LOX-1 siRNA suppresses IL-4 in OGD-treated microglial cells. OGD-treated microglial cells exhibit significant IL-4 concentration.
*: $P<0.05$.
