# Supplementary material for: LOX-1 mediates inflammatory activation of microglial cells through the p38-MAPK/NF-κB pathways under hypoxic-ischemic conditions
Source: Cell Commun Signal. 2023 Jun 2;21:126. doi: 10.1186/s12964-023-01048-w (PMC10236821; doi:10.1186/s12964-023-01048-w)
Supplement: Supplementary file 9 — Additional file 8: Figure S5. OGD induces Nos2 expression. LOX-1 siRNA suppresses Nos2 expression in OGD-treated microglial cells. **P < 0.01. [file 12964_2023_1048_MOESM8_ESM.pdf]

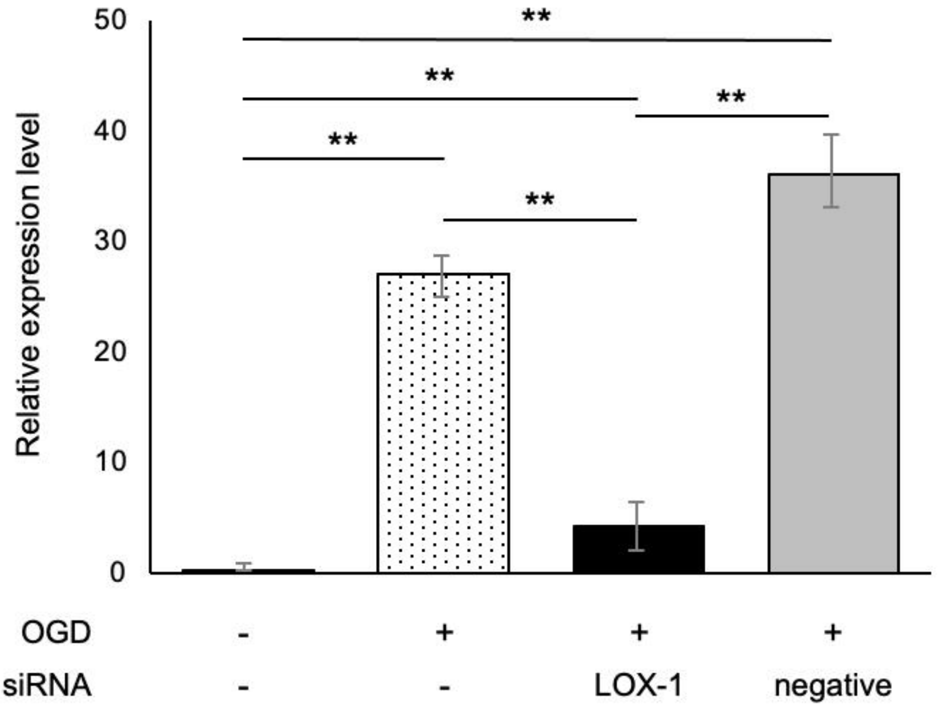

Supplementary Fig. 5. OGD induces Nos2 expression. LOX-1 siRNA suppresses Nos2 expression in OGD-treated microglial cells.

**: *P*<0.01.
