# Supplementary material for: LOX-1 mediates inflammatory activation of microglial cells through the p38-MAPK/NF-κB pathways under hypoxic-ischemic conditions
Source: Cell Commun Signal. 2023 Jun 2;21:126. doi: 10.1186/s12964-023-01048-w (PMC10236821; doi:10.1186/s12964-023-01048-w)
Supplement: Supplementary file 10 — Additional file 9: Figure S6. LOX-1 suppression in OGD-treated microglial cells changes the inflammation-related gene expression pattern. Primary microglial cells express anti-inflammatory gene transcripts. In contrast, OGD-treated microglial cells expressed inflammatory gene transcripts. However, LOX-1 siRNA shifts the M2-transcription pattern. [file 12964_2023_1048_MOESM9_ESM.pdf]

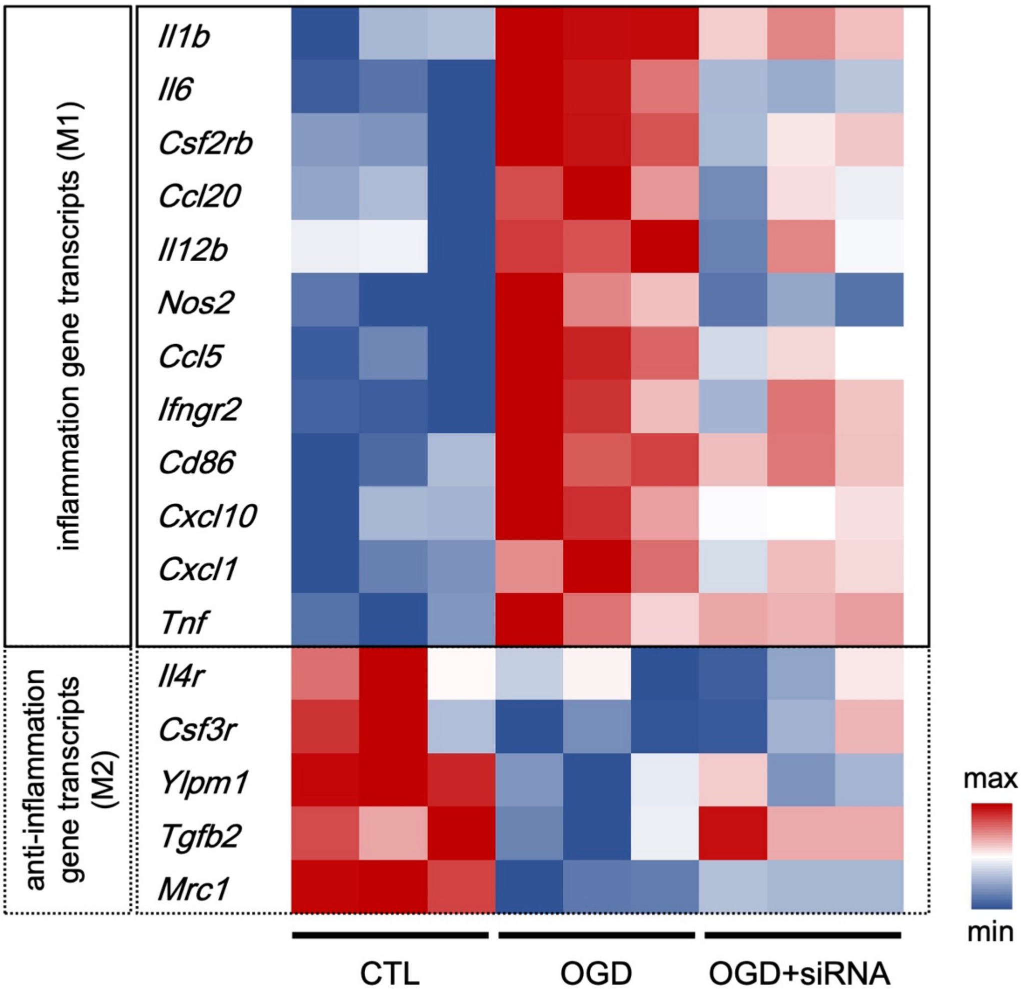

Supplementary Fig. 6. LOX-1 suppression in OGD-treated microglial cells changes the inflammation-related gene expression pattern. Primary microglial cells express anti-inflammatory gene transcripts (M2). In contrast, OGD-treated microglial cells expressed inflammatory gene transcripts (M1). However, LOX-1 siRNA shifts the M2-transcription pattern.
