# Supplementary material for: LOX-1 mediates inflammatory activation of microglial cells through the p38-MAPK/NF-κB pathways under hypoxic-ischemic conditions
Source: Cell Commun Signal. 2023 Jun 2;21:126. doi: 10.1186/s12964-023-01048-w (PMC10236821; doi:10.1186/s12964-023-01048-w)
Supplement: Supplementary file 11 — Additional file 10: Figure S7. p38-MAPK and NF-κB inhibitors show different expression patterns of phosphorylated p38-MAPK in OGD-treated microglial cells. The ratio of phospho/total p38-MAPK was significantly higher level in OGD-treated microglial cells, but was reduced to the control level with SB. However, BAY did not reduce the ratio of phospho/total p38-MAPK. SB; SB203580, BAY; BAY11-7082, *P < 0.05, **P < 0.01. [file 12964_2023_1048_MOESM10_ESM.pdf]

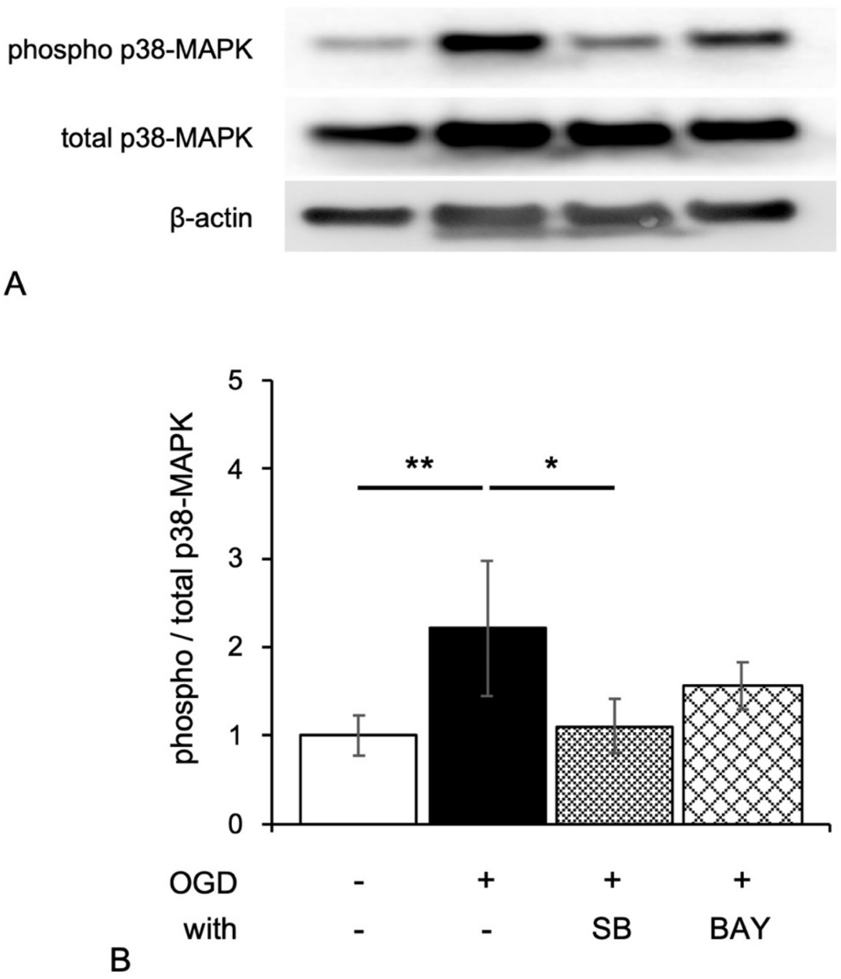

Supplementary Fig. 7. p38-MAPK and NF-κB inhibitors show different expression patterns of phosphorylated p38-MAPK in OGD-treated microglial cells (A). The ratio of phospho/total p38-MAPK was significantly higher level in OGD-treated microglial cells, but was reduced to the control level with SB (B). However, BAY did not reduce the ratio of phospho/total p38-MAPK. SB; SB203580, BAY; BAY11-7082, *; P<0.05, **; P<0.01.
