# Supplementary figures and images for: LOX-1 mediates inflammatory activation of microglial cells through the p38-MAPK/NF-κB pathways under hypoxic-ischemic conditions
Source: Cell Commun Signal. 2023 Jun 2;21:126. doi: 10.1186/s12964-023-01048-w (PMC10236821; doi:10.1186/s12964-023-01048-w)

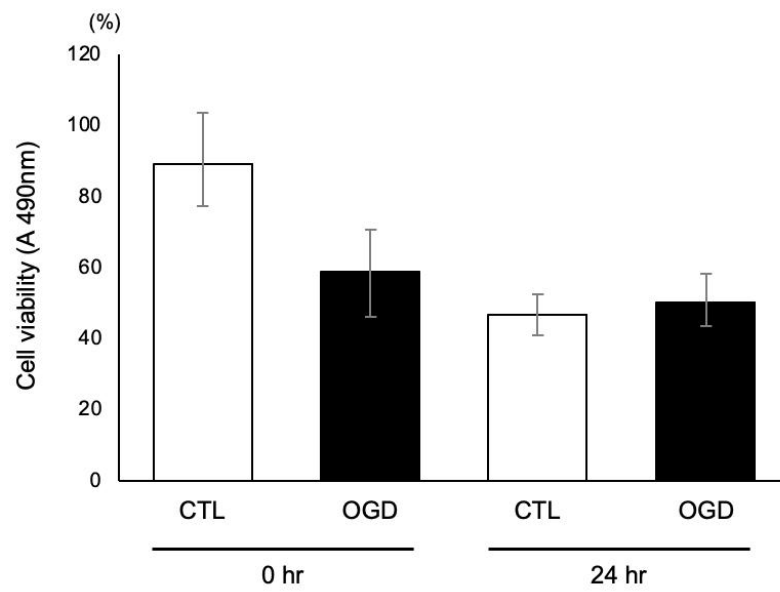

**Supplementary Fig. 8.** Microglial cell viability is not affected by OGD. There are no significant.

Supplement: Supplementary file 12 — Additional file 11: Figure S8. Microglial cell viability is not affected by OGD. There are no significant. [file 12964_2023_1048_MOESM11_ESM.pdf]
