# Supplementary material for: LOX-1 mediates inflammatory activation of microglial cells through the p38-MAPK/NF-κB pathways under hypoxic-ischemic conditions
Source: Cell Commun Signal. 2023 Jun 2;21:126. doi: 10.1186/s12964-023-01048-w (PMC10236821; doi:10.1186/s12964-023-01048-w)
Supplement: Supplementary file 13 — Additional file 12: Figure S9. The chromatin immunoprecipitation assay demonstrated that the both transcription factors of NF-κB and HIF-1α bind to the OLR-1 gene promoter region under the OGD conditions. M; 100-bp ladder marker, 1; I-κBa promoter region as a control, 2; VEGFA promoter region as a control, 3; ③ of NF-κB binding site, 4; ④ of HIF-1α binding site, 5; ⑤ of HIF-1α binding site, 6; ⑥ of NF-κB binding site, D; distilled water. [file 12964_2023_1048_MOESM12_ESM.pdf]

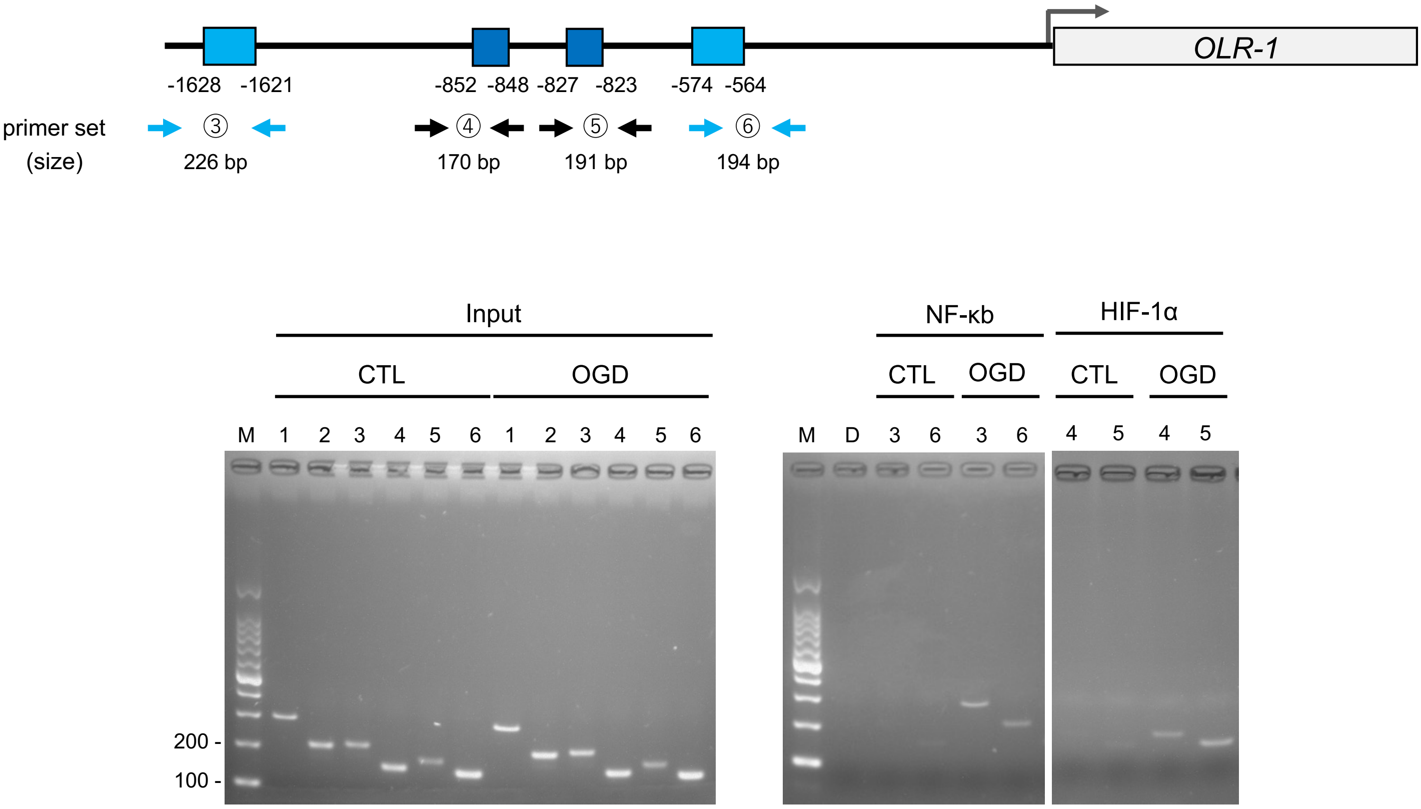

Supplementary Fig. 9. The chromatin immunoprecipitation assay demonstrated that the both transcription factors of NF-κB and HIF-1α bind to the *OLR-1* gene promoter region under the OGD conditions.

M; 100-bp ladder marker, 1; I-κBa promoter region as a control (product size of 299 bp), 2; VEGFA promoter region as a control (220 bp), 3; ③ of NF-κB binding site (226 bp), 4; ④ of HIF-1α binding site (170 bp), 5; ⑤ of HIF-1α binding site (191 bp), 6; ⑥ of NF-κB binding site (194 bp), D; distilled water.
